# Sex-specific DNA methylation signatures of autism spectrum disorder from whole genome bisulfite sequencing of newborn blood

**DOI:** 10.1186/s13293-025-00712-9

**Published:** 2025-04-30

**Authors:** Julia S. Mouat, Nickilou Y. Krigbaum, Sophia Hakam, Emily Thrall, George E. Kuodza, Julia Mellis, Dag H. Yasui, Piera M. Cirillo, Yunin J. Ludena, Rebecca J. Schmidt, Michele A. La Merrill, Irva Hertz-Picciotto, Barbara A. Cohn, Janine M. LaSalle

**Affiliations:** 1 Department of Medical Microbiology and Immunology, School of Medicine, University of California, Davis, CA USA; 2 Perinatal Origins of Disparities Center, University of California, Davis, CA USA; 3 Genome Center, University of California, Davis, CA USA; 4 MIND Institute, University of California, Davis, CA USA; 5 Child Health and Development Studies, Public Health Institute, Berkeley, CA USA; 6 Department of Public Health Sciences, University of California, Davis, CA USA; 7 Department of Environmental Toxicology, University of California, Davis, CA USA; 8 Environmental Health Sciences Center, University of California, Davis, CA USA

**Keywords:** Autism spectrum disorder, DNA methylation, Whole genome bisulfite sequencing, Newborn blood, Newborn dried blood spots, Sex differences, Female protective effect, Epigenetics, X chromosome, Differentially methylated regions

## Abstract

**Background:**

Autism spectrum disorder (ASD) is a group of neurodevelopmental conditions currently diagnosed through behavioral assessments in childhood, though neuropathological changes begin *in utero*. ASD is more commonly diagnosed in males, a disparity attributed to both biological sex differences and diagnostic biases. Identifying molecular biomarkers, such as DNA methylation signatures, could provide more objective screening for ASD-risk in newborns, allowing for early intervention. Epigenetic dysregulation has been reported in multiple tissues from newborns who are later diagnosed with ASD, but this is the first study to investigate sex-specific DNA methylation signatures for ASD in newborn blood, an accessible and widely banked tissue.

**Methods:**

We assayed DNA methylation from newborn blood of ASD and typically developing (TD) individuals (discovery set *n* = 196, replication set *n* = 90) using whole genome bisulfite sequencing (WGBS). Sex-stratified differentially methylated regions (DMRs) were assessed for replication, comparisons by sex, overlaps with DMRs from other tissues, and enrichment for biological processes and SFARI ASD-risk genes.

**Results:**

We found that newborn blood ASD DMRs from both sexes significantly replicated in an independent cohort and were enriched for hypomethylation in ASD compared to TD samples, as well as location in promoters, CpG islands, and CpG island shores. By comparing female to male samples, we found that most sex-associated DMRs in TD individuals were also found in ASD individuals, alongside additional ASD-specific sex differences. Female-specific DMRs were enriched for X chromosomal location. Across both sexes, newborn blood DMRs overlapped significantly with DMRs from umbilical cord blood and placenta but not post-mortem cerebral cortex. DMRs from all tissues were enriched for neurodevelopmental processes (females) and known ASD genes (both sexes).

**Conclusions:**

Overall, we identified and replicated a sex-specific DNA methylation signature of ASD in newborn blood that supported the female protective effect and highlighted convergence of epigenetic and genetic signatures of ASD in newborns. Despite the study’s limitations, particularly in female sample sizes, our results demonstrate the potential of newborn blood in ASD screening and emphasize the importance of sex-stratification in future studies.

**Supplementary Information:**

The online version contains supplementary material available at 10.1186/s13293-025-00712-9.

## Abstract (plain language summary)

Autism spectrum disorder (ASD) is a group of conditions that affect the developing brain during pregnancy but is not diagnosed until childhood based on a behavioral test. Males are diagnosed with ASD more often than females, likely due to both biological sex differences and cultural biases. A molecular/biological test that can predict ASD risk in newborns would provide a more consistent and objective diagnosis and earlier interventions. DNA methylation marks help regulate our genes to respond to a changing environment. DNA methylation differences in ASD have been previously reported, but sex differences in ASD newborn blood have not been explored. We discovered ASD DNA methylation differences through whole-genome bisulfite sequencing in a discovery cohort (*n* = 196) and replicated findings in another (*n* = 90). ASD DNA methylation differences were strongest when samples were separated by sex. Females showed stronger and more consistent DNA methylation patterns related to ASD, supporting the “female protective effect,” which hypothesizes that females may require more substantial genetic or environmental changes to develop ASD. Female-specific DNA methylation changes in ASD were notably enriched on the X chromosome. We also observed overlap between ASD-related DNA methylation changes in newborn blood and other tissues (cord blood, placenta, and brain cortex), with significant enrichment for genes involved in neurodevelopment and known ASD-risk genes. These results highlight the potential utility of newborn blood in ASD screening and the importance of separating analysis by sex in future studies.

**Supplementary Information:**

The online version contains supplementary material available at 10.1186/s13293-025-00712-9.

## Highlights

Sex-specific DNA methylation signatures of ASD were detected in newborn dried blood spot samples.

Females with ASD showed a stronger DNA methylation signature than males, consistent with a “female protective effect” hypothesized to explain the ASD sex ratio.

Females with ASD showed an enrichment for differentially methylated genes on the X chromosome.

In females, three genes differentially methylated in ASD replicated across four different tissues (newborn blood, cord blood, placental, post-mortem cortex): *BCOR* (chrX), *GALNT9* (chr12), and *OPCML* (chr11).

**Supplementary Information:**

The online version contains supplementary material available at 10.1186/s13293-025-00712-9.

## Introduction

Autism spectrum disorder (ASD) is a group of neurodevelopmental conditions diagnosed by functional impairments in social communication and interactions, combined with elevated restrictive interests and repetitive behaviors [1]. Although neuropathology has been shown to begin during gestation [2–7], ASD is typically not diagnosed until early childhood (~ age 3–4) by behavioral observation and clinical assessment [8]. Males are diagnosed with ASD approximately four times more frequently than females. This difference may, in part, reflect an underdiagnosis of true cases in females [9] due to diagnostic definitions and tools that primarily consider symptoms more common in males [10–13]. However, a liability threshold “female protective effect” has been posited to explain the higher number of likely pathogenetic genetic mutations in females, compared to males diagnosed with ASD [14]. For children diagnosed with ASD, intensive behavioral intervention programs have been associated with better outcomes, including higher IQ and improved adaptive behaviors such as communication, daily living skills, socialization, and motor skills [15, 16]. However, these programs are most effective when begun at an early age, including as young as nine months [17], meaning that children are often diagnosed too late in life to fully benefit. Therefore, objective screening tools that use biological measures to identify newborns at increased risk of ASD may help with earlier diagnosis and improved outcomes for many children, particularly females.

Insights to biomarker discovery may be gained by researching newborn tissues and biological markers that have been previously under-studied, such as epigenetic marks from newborn blood. Several studies have shown that the epigenetic mark of DNA methylation can reflect genetic dysregulation in individuals with ASD, even prior to diagnosis [18–23]. DNA methylation patterns are established *in utero*, during which time its landscape is influenced by genetic heritability as well as environmental factors and gene-environment interactions. This triad – genetics, environmental factors, and gene-environment interactions -- also lies at the intersection of etiology for ASD, giving DNA methylation the unique advantage of reflecting multiple early influences of ASD pathology. Intriguingly, ASD studies investigating perinatal tissues such as placenta and umbilical cord blood have shown dysregulated DNA methylation over loci involved in neurodevelopment, demonstrating the promise of perinatal tissues as a surrogate for the brain [18–22]. In contrast to cord blood and placenta, newborn blood is accessible and widely banked through newborn dried blood spot (NDBS) programs across several states.

**Fig. 1:**
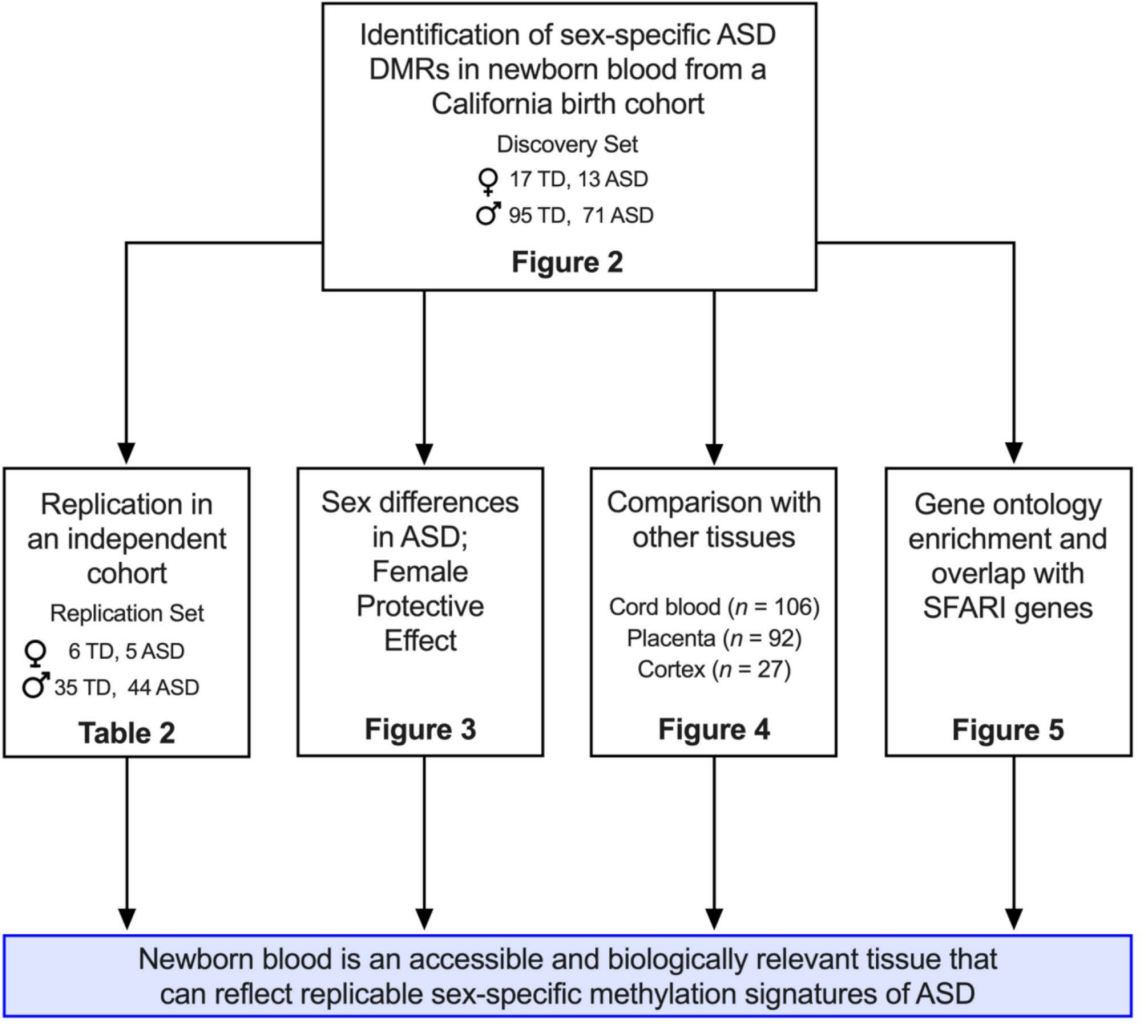
Flowchart of the study

Here, we describe the identification of the first sex-specific DNA methylation signatures of ASD from newborn blood using a multi-staged approach (Fig. 1). We used NDBS from children who were later diagnosed with ASD and sex- and age-matched typical developing (TD) children from a California birth cohort (*n* = 196). Following whole-genome bisulfite sequencing (WGBS), which provides the advantage of covering the entire genome, we identified sex-combined and sex-stratified ASD vs. TD differentially methylated regions (DMRs). From these DMRs, we identified a sex-specific DNA methylation signature of ASD in newborn blood that was highly replicated in an independent case-control cohort (*n* = 90), demonstrated epigenetic support for the female protective effect, overlapped with ASD DMRs from other tissues (previously published cord blood [18], placenta [22], and cortex [24]), and was enriched for neurologic-related biological processes and known ASD-risk genes. Overall, our findings show that newborn blood is an accessible and biologically relevant tissue that can reflect sex-specific DNA methylation signatures of ASD, providing potential insights for biomarker development.

## Materials and methods

### Study population

We investigated DNA methylation signatures from ASD and TD individuals using samples from two cohorts of newborn blood (discovery set: 30 females (17 TD, 13 ASD), 166 males (95 TD, 71 ASD); replication set: 11 females (6 TD, 5 ASD), 79 males (35 TD, 44 ASD), as well as umbilical cord blood (32 females (17 TD, 15 ASD), 74 males (39 TD, 35 ASD)), placenta (30 females (15 TD, 15 ASD), 62 males (31 TD, 31 ASD)), and cortex (10 females (6 TD, 4 ASD), 17 males (5 TD, 12 ASD)). The DNA methylation patterns from cord blood [18], placenta [22], and frontal cortex (Brodmann area 9 grey matter) [24] samples have been previously published and are available in the Gene Expression Omnibus with accession numbers GSE140730 (cord blood), GSE178206 (placenta), GSE81541 (cortex) and GSE119981 (cortex). We used the “discovery” data set samples from the cord blood and placenta publications [18, 19]. Newborn blood, cord blood, and placenta samples were all collected at time of birth, and the post-mortem cortices were collected from individuals aged 4–56 years at the time of death. All samples were collected from different individuals and cohorts, except for placenta and cord blood, which had considerable overlap.

Discovery newborn blood samples were obtained through the Child Health and Development Studies (CHDS), a hospital-based birth cohort that enrolled > 20,000 pregnant women at the Kaiser Foundation Health Plan in Oakland, CA from 1959 to 1967. Over 98% of eligible women enrolled in the study, therefore reflecting the socio-demographics of the Oakland area at the time and making CHDS a more racially diverse cohort than most, with > 40% of the selected samples belonging to a racial or ethnic category other than non-Hispanic white (Supplemental Table 1). Follow-up studies have collected information and biological samples from the children and grandchildren of the original cohort. We matched the CHDS database with the California Department of Developmental Services (DDS) to identify grandchildren of the original CHDS cohort who have been diagnosed with ASD. Registration of ASD within the California DDS requires diagnosis within a regional center. TD individuals were chosen from CHDS grandchildren without any DDS diagnoses, matched according to sex and birth year to the ASD individuals. These ASD cases and TD controls were then matched to the California Biobank Program for available NDBS samples, resulting in 196 deidentified NDBS for the discovery group. Replication newborn blood samples were obtained from participants of the CHARGE case-control study in Northern California, which has been previously described [25]. The ASD diagnoses are confirmed at the UC Davis MIND Institute, using the Autism Diagnostic Interview–Revised (ADI-R) and the Autism Diagnostic Observation Schedules ADOS) [26, 27]. For both studies, TD participants are those within the same cohort who did not have any neurodevelopmental disorder listed in the DDS.

Cord blood and placenta samples were obtained through the MARBLES study, an enriched-likelihood cohort that enrolls pregnant women in Northern California who already have a biological child with ASD. The TD participants from MARBLES are clinically defined as non-autistic, non-developmentally delayed, having undergone the same behavioral and cognitive assessments as the ASD cases: ADI-R and ADOS. The human cerebral cortex samples from Brodmann Area 9 were obtained from the National Institute of Child Health and Human Developmental Brain and Tissue Bank for Developmental Disorders at the University of Maryland [24].

### DNA extraction and whole genome bisulfite sequencing

DNA methylation from all samples was assayed using WGBS. Discovery NDBSs were each punched into six 4 mm punches, which were stored at − 80 C. The samples were randomized by ASD diagnosis and sex and then grouped into ten batches for DNA extraction. Two DNA extractions were performed per sample, each using three 4 mm punches, using the GenTegra Complete DNA Recovery Kit (GenTegra, Pleasanton, CA, USA) and the QIAamp Micro Kit (Qiagen, Hilden, Germany), based on Additional File 2 Protocol GQ from Ghantous et al., 2014 [28]. The two isolations from each sample were combined into a single 1.7 µL tube and ethanol precipitation was used to clean the samples. The DNA was re-eluted in 30 µl nuclease-free water and quantified by Qubit. DNA purity was assessed by Nanodrop 260/230 and 260/280 ratios. DNA samples were sonicated to a fragment size of ~ 350 bp with a peak power of 175, duty of 10%, and 200 cycles/burst for 47 s using 15 µl DNA and 40 µl EB. Sonicated DNA was cleaned and concentrated with gDNA clean and concentrator columns (Zymo Research, Irvine, CA, USA), eluted in 25 µl EB, and re-quantified by Qubit. The maximum mass of DNA for each sample, up to 100 ng, was then bisulfite converted using the Zymo EZ DNA Methylation Lightning Kit. Illumina sequencing libraries were prepared using the ACCEL-NGS MethylSeq DNA library kit (Swift Biosciences, Ann Arbor, MI, USA), with 6 cycles of indexing PCR (7 cycles for lower input samples). The libraries were pooled and sequenced across 13 lanes of NovaSeq S4 flow cells (Illumina, San Diego, CA, USA) for 150 bp paired end reads with a 5% PhiX spike-in to generate ~ 200 million reads (~ 8-12x coverage, 60 Gb) per sample. Replication NDBSs were processed and sequenced the same as discovery, except that only one DNA isolation was performed per sample using 2–4 4 mm punches and ethanol precipitation was not used to clean the DNA before library preparation.

Cord blood, placenta, and cerebral cortex samples were processed as previously described [18, 22, 24]. Briefly, DNA was extracted from cord blood samples with the Qiagen Puregene Blood kit, and from placental and cortex samples with the Qiagen Puregene kit. DNA from all three tissues was bisulfite converted with the Zymo EZ DNA Methylation Lightning kit. Illumina sequencing libraries were prepared from cord blood and placental samples using the Illumina TruSeq DNA Methylation kit with indexed PCR primers and a 14-cycle PCR program, and from cortex samples as previously described [24]. Cord blood and placental samples were sequenced at 2 per lane with 150 bp paired-end reads and spiked-in PhiX DNA on the Illumina HiSeq X. Cortex samples were sequenced at one per lane with 100 bp single-end reads on the Illumina HiSeq 2000.

### Descriptive statistics for the study population

The descriptive statistics compared demographic, parental, and prenatal factors stratified by ASD. This included both categorical and continuous variables, using statistical methods to test for differences between groups. Key variables such as sex, birth year, parental education, race/ethnicity, and insurance status were examined. For continuous variables such as maternal age, a two-sample t-test was used to compare means. For categorical variables such as maternal education, chi-squared tests of independence or Fisher’s exact (frequency count < 5) were used to assess associations between the variable of interest and the stratification group. This analysis was performed with R v4.4.1 and SAS v9.4 (Enterprise Edition).

### Sequence alignment and quality control

FASTQ files for each sample were merged across lanes using FASTQ_Me [29] and aligned to the hg38 genome using CpG_Me [30] with the default parameters [31–34]. The alignment pipeline includes trimming adapters and correcting for methylation bias, screening for contaminating genomes, aligning to the reference genome, removing PCR duplicates, calculating coverage and insert size, and extracting CpG methylation to generate a cytosine report (CpG count matrix) and a quality control report.

### Determination of sex

Sex was defined in this study as genotypic sex, whereby those with two X chromosomes were defined as females and those with one X and one Y chromosome were defined as males. Our sample sets did not include any individuals that had other combinations of sex chromosomes. We determined genotypic sex for each sample by calculating the ratio of sex chromosomes from WGBS reads using the SexChecker pipeline (https://github.com/hyeyeon-hwang/SexChecker). The gender identity of study participants is not known to us, and we acknowledge that gender and genotypic sex may not align for some participants. With this manuscript’s goal of better understanding ASD pathogenesis and identifying biomarkers that may be used for newborns, we feel that genotypic sex is an appropriate measure.

### Global methylation analyses

Global DNA methylation for each sample was calculated as the total number of methylated CpG counts divided by the total number of CpG counts from all CpGs included in the DMRichR analysis (filtering described below). Differences in global methylation across tissues were tested with one-way ANOVA with Tukey’s multiple comparisons while differences across sexes and ASD diagnoses were tested with two-way ANOVA with Fisher’s Least Significant Difference using GraphPad Prism 10.0.3.

### Differentially methylated regions

Sex-combined and sex-stratified ASD vs. TD DMRs were identified for each tissue (discovery newborn blood, replication newborn blood, cord blood, placenta, cortex), as well as female vs. male DMRs for discovery newborn blood using DMRichR (https://github.com/ben-laufer/DMRichR) [35–37]. ASD vs. TD DMRs included autosomal and sex chromosomes while the female vs. male DMRs included only autosomes. We defined regions as sections of the genome that have at minimum 5 CpGs and a maximum gap of 1000 base pairs, with the “universe” for each comparison described as all CpGs included in the analysis. All sex-combined analyses were adjusted for sex, and cortex analyses were additionally corrected for age at time of death. We did not adjust DMRs for cell types due to large inconsistencies across reference datasets [35] and our previous finding that adjustment for cell types did not significantly influence the calling of ASD DMRs in cord blood [18].

Because each tissue and sex contained a different number of samples, we normalized the analyses by adjusting the percent of samples per group that must have at least 1x coverage over a certain CpG site for its inclusion in the analysis. Datasets with fewer samples tend to produce a greater number of DMRs (many of which may be false positives), and datasets with many samples may produce very few (< 10) DMRs (which may exclude many real DMRs). To adjust for this, we required all datasets to have the same number of samples covered over a certain CpG for its inclusion in the analysis, which results in a higher percentage of samples for datasets with fewer samples, thereby decreasing the number of DMRs and vice versa for large datasets. Because cortex had the fewest number of samples (*n* = 27), we set perGroup = 1 for all cortex analyses, and for all other tissues, calculated perGroup as 27/*n* so that each comparison required 27 samples to have coverage over a given CpG for its inclusion in the DMR analysis. Otherwise, we used default DMRichR parameters. We used a threshold of permutation *p*-value < 0.05 for DMR identification. Because of the highly correlated patterns of DNA methylation patterns in newborn blood [38], we did not adjust for multiple hypothesis tested but instead used replication to reduce false positives [39].

### Down-sampling males for discovery newborn blood DMR analysis

Because there were a greater number of males than females in the study, discovery newborn blood males were down sampled to match the number of females in order to evaluate the DMRs in each sex when sample sizes were equal. A random number generator was used to select 30 male samples (17 TD, 13 ASD to match the females) for each batch. The “perGroup” value matched for females and down sampled male batches.

### Principal component analysis

DMRs were also visualized using principal component analysis (PCA). Smoothed methylation values over nominally significant (*p* < 0.05) DMRs from each comparison were obtained using the “getMeth” function in the bsseq R package v1.36.0. PCA was performed using the “prcomp” function in the stats R package with centering to zero and scaling to unit variance. PCA results were plotted using the “autoplot” function in ggfortify v0.4.16 using R v4.3.1 with an ellipse indicating the 95% confidence level for each group, assuming a multivariate normal distribution.

### Proposed models for sex differences in ASD

The discovery newborn blood dataset was used to investigate three proposed models for sex differences in ASD [40]: (1) the multifactorial liability model, including the female protective effect, whereby female-specific protective factors (and/or male-specific vulnerability factors) shift females further away from the liability threshold [14]; (2) the extreme male brain theory, whereby all individuals with ASD have a shift towards male phenotypes [41]; and (3) the gender incoherence theory, whereby ASD attenuates sex differences present in TD individuals [42].

### Chromosome enrichment analysis

EnrichR [43–45] was used to test discovery and replication newborn blood ASD vs. TD DMRs for enrichment across individual chromosomes. The inputs were DMRs mapped to the nearest gene on hg38 with the background set to the “universe” for each DMR comparison, as described above.

### Differentially methylated region overlaps by genomic location and gene name

ASD DMRs were overlapped by genomic location using the Genomic Ranges and RegioneR R packages. Because of the tiny proportion of the genome taken up by DMRs, they are highly unlikely to overlap by chance. The permTest function in the RegioneR package, v1.32.0, was used to calculate significance of overlaps by genomic region as well as mean distance between DMRs of two comparisons. The “genome” was defined as the intersection of all regions included in the two individual analyses. Regions were randomly resampled and tested with 10,000 permutations. For overlap by gene name, ASD DMRs were mapped to the closest gene on the hg38 genome using the “oneClosest” rule of the Genomic Regions Enrichment of Annotations Tool (GREAT) [46]. Statistics for pairwise comparisons of gene name overlaps were calculated using the Fisher’s Exact Test in the GeneOverlap R package, v1.36.0, in which the “genome” was defined as the intersection of all regions included in the two analyses, mapped to the nearest gene. Numbers of DMR gene name overlaps were visualized using the UpSetR R package v1.4.0 [47].

### Gene ontology term overlaps

Gene ontology (GO) enrichment of DMRs from each ASD vs. TD comparison were identified with GOfuncR [48], which performs permutation based enrichment testing for the genomic coordinates of the DMRs relative to those of the background regions. Using the sex-stratified DMRs from each tissue, GO terms related to biological processes, molecular functions, and cellular components that had a *p*-value < 0.2 were intersected across all tissues. The overlapping biological processes were graphed by their -log(*p*-value) using GraphPad Prism v 10.0.3, ordered by highest to lowest average -log(*p*-value) across tissue.

### SFARI gene enrichment

DMRs mapped to genes from all comparisons were overlapped with genes listed in the Simons Foundation Autism Research Initiative (SFARI) database, release date 3/28/2024 [49]. The significance of the overlaps was calculated with the Fisher’s exact test with the background defined as the “universe” for each DMR comparison, as described above.

## Results

### Study subject characteristics

In this study, we investigated WGBS DNA methylation signatures from ASD and TD individuals using newborn blood. We used a discovery cohort of 196 samples [30 females (17 TD, 13 ASD), 166 males (95 TD, 71 ASD)] from grandchildren of the CHDS birth cohort, and replicated our results in an independent cohort of 90 samples [11 females (6 TD, 5 ASD), 79 males (35 TD, 44 ASD)] from the CHARGE case-control cohort. Because ASD is more frequently diagnosed in males and the male:female ratio is decreasing over time, TD controls were matched for both sex and birth year. From the additional information available from birth certificates, only insurance coverage and paternal race/ethnicity were significantly different between ASD and TD within the CHDS cohort, with ASD cases having a higher rate of insurance (91.7% vs. 75.9%, *p* < 0.005), and more Hispanic but fewer non-Hispanic white and non-Hispanic black fathers (*p* < 0.02) than TD (Additional File 1, Table S1). There were no significant differences in the ASD vs. TD samples in the CHARGE replication cohort and both cohorts were demographically diverse (Additional File 1, Tables S1-2). In both cohorts, global methylation was significantly lower in males than in females but did not differ between ASD and TD samples in either sex (Additional File 2, Figure S1).

### Discovery newborn blood DMRs

**Fig. 2:**
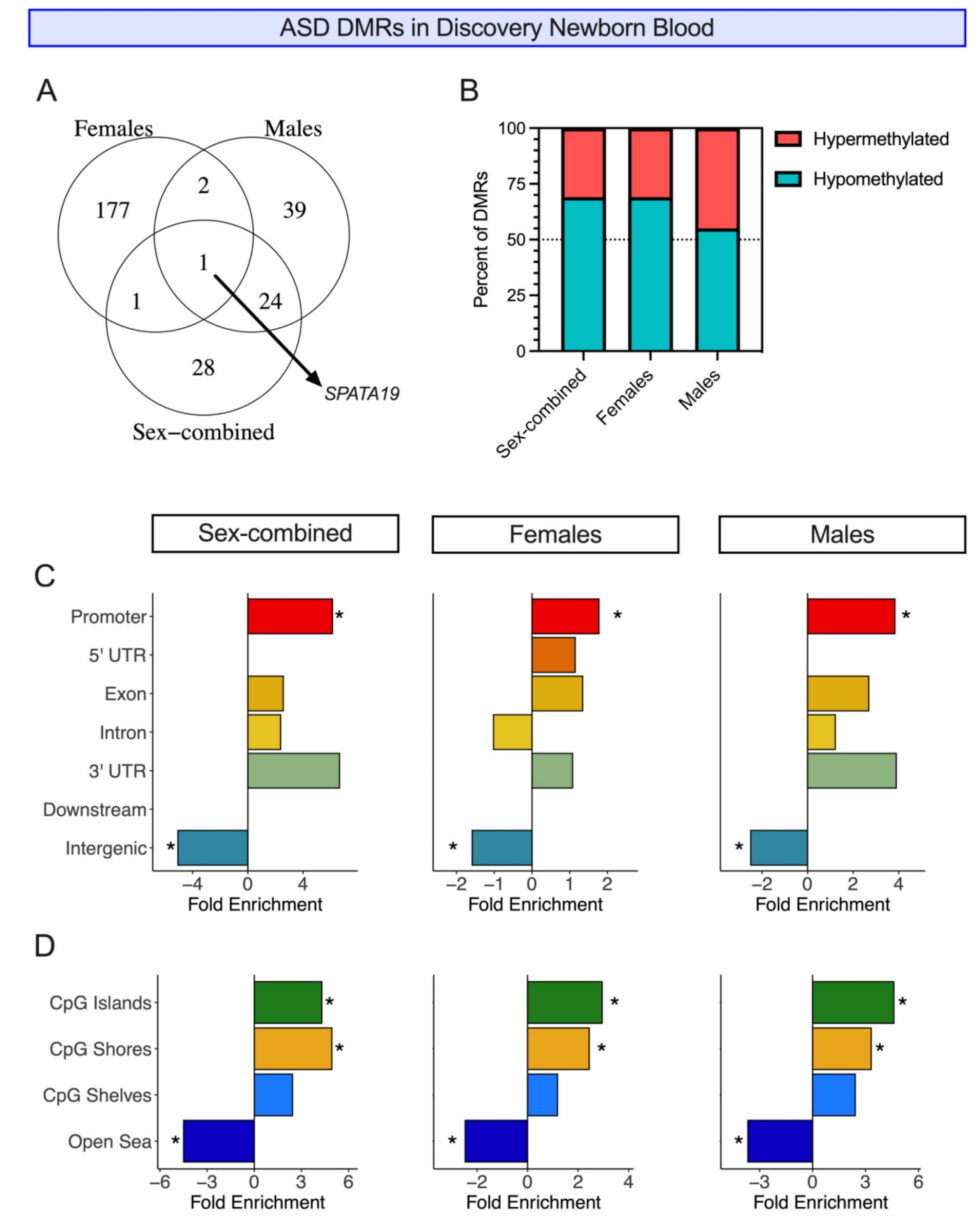
ASD DMRs in discovery newborn blood. **(A)** Overlaps between ASD DMRs mapped to the nearest gene on the hg38 genome from sex-combined, females only, and males only comparisons. **(B)** The percent of DMRs (permutation *p* < 0.05) that are hypo- or hypermethylated in ASD compared to TD samples in sex-combined, females only, and males only comparisons. **(C)** Genic and **(D)** CpG context enrichments of DMRs (permutation *p* < 0.05) from sex-combined, females only, and males only comparisons. DMRs were compared to background regions for each comparison and significance was determined by the Fisher’s test and FDR correction. * = q < 0.05

We called DMRs for ASD vs. TD samples in discovery newborn blood, identifying 59 DMRs in the sex-combined comparison (corrected for sex), 185 in females only, and 99 in males only (Table 1; Additional File 1, Table S3-5; Additional File 2, Figure S2). Upon mapping DMRs to the closest gene on the hg38 genome (54 DMR genes in the sex-combined comparison, 181 in females, 66 in males) and overlapping gene names across the three comparisons, we found that only one DMR gene (*SPATA19*) was present in all comparisons. Of the 181 DMR genes identified only in females, 177 (97.8%) were unique to that comparison. In contrast, 59% (39/66) of DMR genes identified only in the males overlapped with 51.8% (28/54) of those in the sex-combined, primarily due to the large overlap between males only and sex-combined DMR genes (Fig. 2A). By genomic location, two DMRs overlapped across females and males, but one of these was methylated in opposite directions in the two sexes, therefore not appearing in the sex-combined analysis.

All comparisons showed a signature of hypomethylation in ASD compared to TD samples, but this was more pronounced in the females-only and sex-combined comparisons, where 69% of DMRs were hypomethylated, in contrast to 55% in males (Fig. 2B). There was also consistency in the genic and CpG contexts across all comparisons, with significant enrichment for DMRs being in promoters as well as CpG islands and shores in the sex-combined, females-only, and males-only analyses (Fig. 2C-D).

Because there were over five times as many males as females in our discovery newborn blood dataset, we also evaluated the epigenetic signature of ASD in subgroups of males that matched the number of samples in females to evaluate the consistency of male DMRs. We randomly subset the males into subgroups of 30 (13 ASD and 17 TD to match the females) and called ASD vs. TD DMRs for each batch. We saw that the DMRs separated samples by ASD diagnosis better by hierarchical clustering and PCA in each of the five subgroups of males than when all males were included in a single analysis, matching the females and likely reflecting heterogeneity across male samples (Additional File 2, Figure S3). Despite this heterogeneity, DMR genes from all five male subgroups significantly overlapped with one another as well as with the DMR genes identified from all 166 males (Additional File 2, Figure S4), giving us confidence to continue our analysis with the original DMRs identified from all discovery males.

**Table 1:** ASD vs. TD DMRs in discovery newborn blood

Comparison	*n*	*n* (ASD)	*n* (TD)	# DMRs	# DMR Genes
**Discovery Newborn Blood: CHDS birth cohort**					
Sex-combined	196	84	112	59	54
Females	30	13	17	185	181
Males	166	71	95	99	66
**Replication Newborn Blood: CHARGE case-control study**					
Sex-combined	90	48	41	67	64
Females	11	5	6	5204	4017
Males	79	44	35	189	172

### Newborn blood ASD DMR genes replicated in sex-stratified comparisons

We then evaluated the consistency of epigenetic dysregulation in newborn blood by calling ASD DMRs in an independent cohort, finding 67, 5204, and 189 DMRs in sex-combined, females only, and males only comparisons, respectively (Table 1; Additional File 1, Tables S6-8; Additional File 2, Fig. S3). When we mapped DMRs to the closest gene on the hg38 genome and overlapped gene lists from discovery and replication analyses, we found significant replication in females-only and males-only comparisons (*p* < 0.05) (Table 2; Additional File 1, Tables S9-10). As a more stringent comparison than gene name overlap, we also compared DMRs from the two cohorts by genomic location. Very few overlaps would be expected by chance since DMRs cover a very small fraction of the genome and indeed, there were fewer overlaps than by gene name in all comparisons (1 in sex-combined, 12 in females only, and 4 in males only). However, all were significant (permutation *p*-value < 0.05) (Additional File 1, Tables S9-10). We also analyzed whether, given a DMR in the discovery group and its closest DMR by location in the replication group, the DMRs were closer together in the genome than would be expected by random chance and found that this was true for all comparisons (permutation *p*-value = 0.0001) (Additional File 1, Table S9; Additional File 2, Figure S6).

**Table 2:** Replication of newborn blood ASD vs. TD DMR genes

Comparison	Discovery Set: Number of DMR Genes	Replication Set: Number of DMR Genes	Overlap	Percent Overlap	Jaccard Index	Odds Ratio	*p* value
Sex-combined	54	64	1	1.85%	0.0085	4.55	0.203
Females	181	4017	87	48.07%	0.021	2.58	**4.79E-10**
Males	66	172	7	10.61%	0.030	10.97	**8.26E-06**

### Discovery newborn blood DMRs show support for the female protective effect

**Fig. 3:**
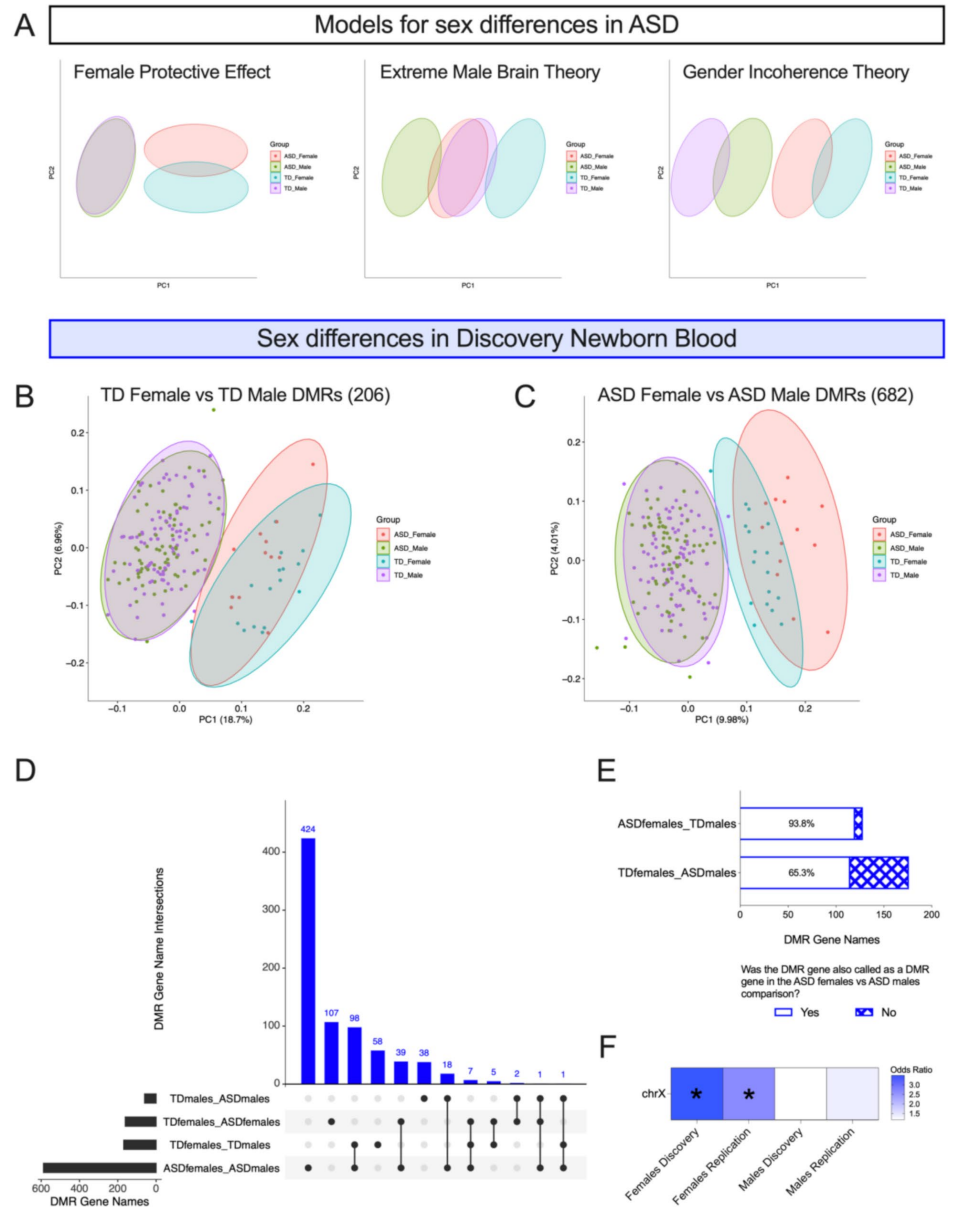
Discovery newborn blood DMRs display female protective effect. **(A)** PCA models reflecting different theories for sex differences in ASD. PCA of smooth methylation values in discovery newborn blood samples over DMRs from **(B)** TD female vs. TD male comparison and **(C)** ASD female vs. ASD male comparison. **(D)** UpSet Plot of autosomal DMRs intersected by gene name. **(E)** Bar plot of ASD female vs. TD male and TD female vs. ASD male comparisons, showing proportion of autosomal DMR genes that were also called as an autosomal DMR gene in the ASD female vs. ASD male comparison. **(F)** Heatmap showing enrichment on the X chromosome from ASD vs. TD DMRs identified from discovery and replication newborn blood females and males. *q < 0.05

Because only three ASD DMR genes overlapped between females and males, we wanted to further explore potential sex differences in our discovery newborn blood dataset. The skew in ASD diagnoses towards males has led to several theoretical models for neurobiological sex differences in ASD [40], including, (1) the multifactorial liability model, including the female protective effect [14]; (2) the extreme male brain theory [41] and; (3) the gender incoherence theory [42] (Fig. 3A). We evaluated whether there was epigenetic support for these theories in newborn blood.

We first identified autosomal DMRs between females and males within TD (TD females vs. TD males) (Table 3) and within ASD (ASD females vs. ASD males) (Additional File 1, Tables S11-12). To understand how sex differences intersect with the methylation signature of ASD, we performed PCA from smoothed methylation values from all discovery newborn blood samples over the diagnosis-specific female vs. male DMRs and found that ASD males did not separate from TD males in either comparison, while ASD females showed some separation from TD females, though the direction of separation depended on the regions assayed: when smooth methylation was assayed over TD female vs. TD male DMRs (Fig. 3B), the ASD females were shifted towards the males, while the opposite occurred when methylation was assayed over ASD female vs. ASD male DMRs (Fig. 3C). These results are most consistent with models of the female protective effect and do not reflect expected findings for the extreme male brain theory or gender incoherence theory.

We next overlapped autosomal DMR gene names from ASD vs. TD and female vs. male comparisons to better understand the proportion of methylation differences that are common versus unique across sexes and diagnoses (Additional File 1, Table S13). We found that the majority (62.7%; *p* = 2.3E-106) of TD female vs. TD male DMR genes were also found in the ASD female vs. ASD male comparison, with many additional DMRs in the latter comparison (Fig. 3D) (Additional File 2, Figure S7). In addition, of the 128 ASD female vs. TD male DMR genes, 120 (93.8%) were also called as ASD female vs. ASD male DMR genes, indicating that epigenetic sex differences are consistent regardless of ASD diagnosis in the males (Fig. 3E). This was not true when examining the epigenetic impact of ASD diagnosis in females, as only 115/176 (65.3%) of the TD female vs. ASD male comparison DMR genes were also called as ASD female vs. ASD male DMR genes. These results support our previous finding across perinatal tissues that the signature of ASD is more distinct in females than in males, a primary prediction of the multifactorial liability threshold and female protective effect model [40].

**Table 3:** Female vs. male DMRs in discovery newborn blood autosomes

Description	Comparison	*n*	# DMRs	# DMR Genes
Sex differences within TD	TD females vs. TD males	112	206	169
Sex differences within ASD	ASD females vs. ASD males	84	682	588
Sex differences across ASD diagnosis	TD females vs. ASD males	88	209	176
Sex differences across ASD diagnosis	ASD females vs. TD males	108	154	128

### Discovery and replication newborn blood ASD DMRs identified from females are enriched for X chromosome location

Because we saw evidence of the female protective effect from our analysis of autosomal chromosomes, we wondered if methylation differences on the X chromosome were further contributing to the signature of ASD in females. Using both the discovery and replication newborn blood datasets, we tested the ASD vs. TD DMRs identified from females only, males only, and sex-combined comparisons for enrichment across all chromosomes. We found that DMRs identified from females in both cohorts were significantly enriched for X chromosome location (*q* < 0.05), while DMRs identified from males only or sex-combined analyses were not significantly enriched for any chromosome (Fig. 3F) (Additional File 1, Table S14). Consistent with this finding, none of the DMRs derived from the down-sampled male subgroups from the discovery set were enriched for any chromosome. Additionally, of the twelve loci that replicated in females across both newborn blood datasets, three (25%) were located on the X chromosome (Additional File 1, Table S10). These findings indicate that the methylation signature of ASD in females is being driven by both autosomal and X-linked loci. We further asked if the replicated female ASD vs. TD DMR X-linked genes were subject versus escaping X chromosome inactivation based on a published consensus of human XCI status. We saw no evidence for any of the differentially methylated genes being consistent XCI “escapees,” as most were always subject to XCI [6] and the remaining were either not called [3] or discordant [1] (Additional File 1, Table S10) [50].

### Newborn blood ASD DMR genes significantly overlap with DMR genes from umbilical cord blood and placenta

Once we established the sex-specificity of the ASD epigenetic signature in newborn blood, we tested if the ASD vs. TD DMRs overlapped with those identified from other tissues. We called DMRs from previously published umbilical cord blood, placenta, and post-mortem cortex WGBS data from ASD and TD individuals using parameters consistent with those used in the newborn blood analysis (Table 4; Additional File 1, Tables S15-23; Additional File 2, Figure S8). Similar to newborn blood, DMRs were more often hypomethylated than hypermethylated in ASD compared to TD samples in females, while in males, this finding was inconsistent across tissues (Additional File 2, Figure S9). While there was significant overlap between DMRs identified between females and males in each tissue, loci were often methylated in opposite directions in the two sexes (Additional File 1, Table S24).

**Fig. 4:**
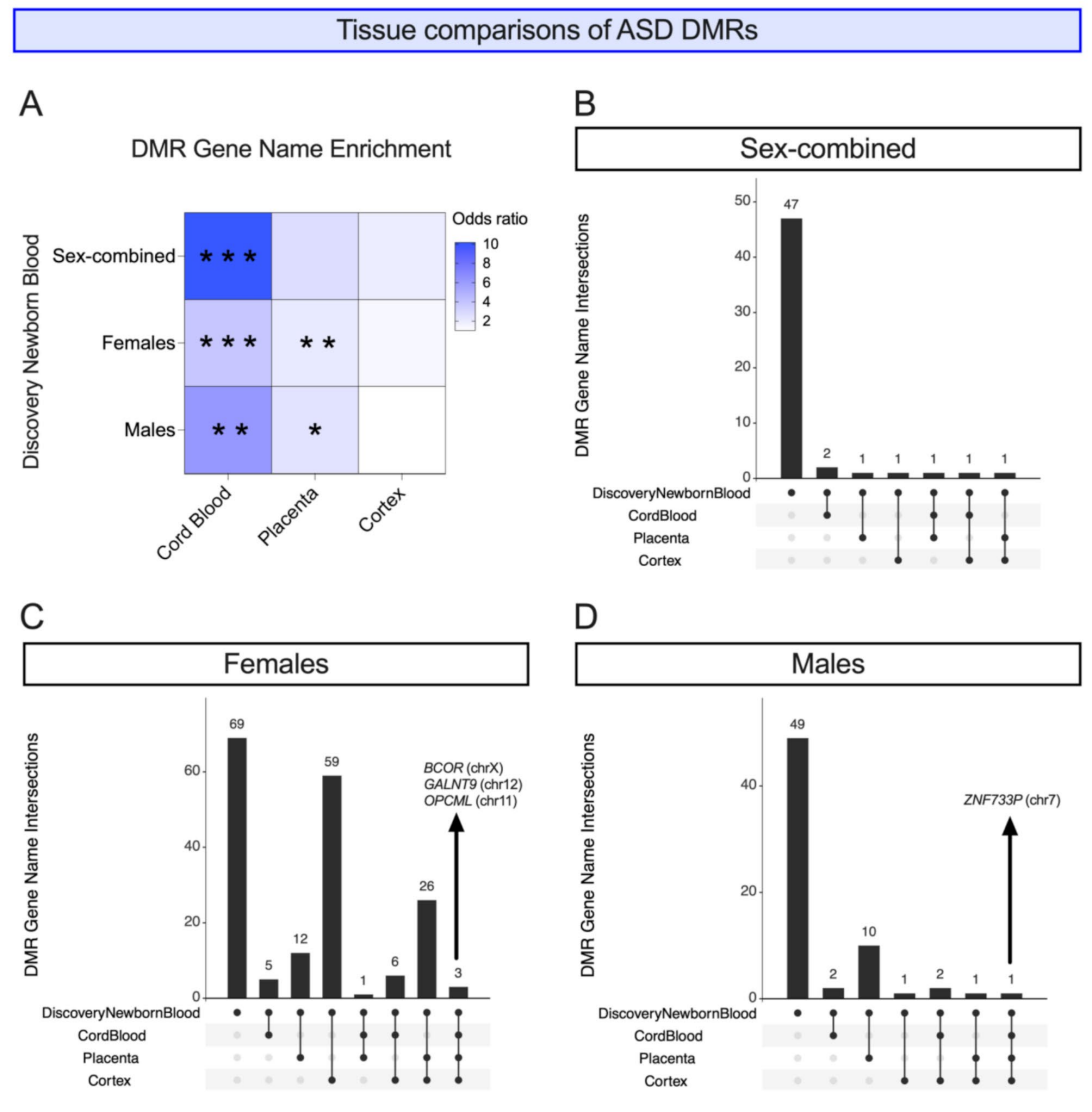
Tissue comparison of ASD DMRs. **(A)** ASD DMR genes from discovery newborn blood were overlapped with those from cord blood, placenta, and cortex from sex-combined, females only, and males only comparisons. Heatmap shows odds ratios of overlaps where darker blue indicates a higher odds ratio. * *p* < 0.05, ** *p* < 0.01, *** *p* < 0.001, **** *p* < 0.0001. UpSet Plots showing numbers of intersections between ASD DMR genes from discovery newborn blood and those from cord blood, placenta, and cortex in **(B)** sex-combined, **(C)** females only, and **(D)** males only comparisons

We mapped DMRs from all analyses to the nearest gene and overlapped gene lists across tissues, finding that discovery newborn blood DMR genes overlapped more than would be expected by chance with those in other tissues. This included umbilical cord blood in females, males, and sex-combined analyses, as well as placenta in females and males, but not cortex in any comparison (Fig. 4A; Additional File 1, Table S25). In the sex-combined comparison, no DMR genes were identified in all four tissues. In females-only, however, three genes mapped to DMRs identified in all four tissues: *BCOR* (chrX), *GALNT9* (chr12), and *OPCML* (chr11) (Fig. 4C; Additional File 2, Figure S10). *BCOR* and *OPCML* were also replicated in newborn blood from females. In males, one gene mapped to DMRs in all tissues: *ZNF733P* (chr7) (Fig. 4D) but was not replicated in the newborn blood replication dataset.

**Table 4:** ASD vs. TD DMRs in cord blood, placenta, and cortex

Comparison	*n*	*n* (ASD)	*n* (TD)	# DMRs	# DMR Genes
**Umbilical Cord Blood: MARBLES enriched-likelihood study (Mordaunt et al., 2020)**					
Sex-combined	106	50	56	127	115
Females	32	15	17	430	413
Males	74	35	39	248	235
**Placenta: MARBLES enriched-likelihood study (Zhu et al.**,** 2022)**					
Sex-combined	92	46	46	557	384
Females	30	15	15	3012	2147
Males	62	31	31	2168	1493
**Post-mortem Cortex: NICHD Brain and Tissue Bank (Vogel Ciernia et al.**,** 2020)**					
Sex-combined	27	16	11	453	435
Females	10	4	6	10,405	6599
Males	17	12	5	1067	984

### ASD DMRs in different tissues are closer together than expected by chance, particularly in females, but are still tissue-specific

As a more stringent comparison than gene name overlap, we overlapped DMRs from newborn blood with other tissues by genomic location. Since DMRs cover a very small fraction of the genome, it is not surprising that there were zero loci that had a DMR in all four tissues in either females or males. However, in females, there were two DMRs that were detected in newborn blood and two other tissues: chr2:130037414–130,037,709 (chr2q21.1 band) in newborn blood (discovery), cord blood, and placenta; and chr20:30899557–30,900,219 (chr20q11.21 band) in newborn blood (discovery and replication), cord blood, and cortex (Additional File 1, Table S26). We calculated the significance of tissue-tissue pairwise overlaps by genomic location and found that, in females, the overlap of newborn blood DMRs was significantly higher than expected by chance versus cord blood (*p* = 9.99E-5) and placenta (*p* = 0.048) (Additional File 1, Table S27). In males, no overlaps between any two tissues were significant.

As the likelihood of DMRs overlapping by genomic locations across analyses is very low, we also tested whether DMRs identified in different tissues were closer together on the genome than would be expected by chance. We found that for all pairwise tissue comparisons of DMRs identified from females, the mean distance of a DMR in newborn blood was closer to the nearest DMR in all other tissues than would be expected by chance (all permutation *p*-values < 0.001) (Additional File 1, Table S27; Additional File 2, Figure S11). In males, ASD DMRs from newborn blood were closer by chance with DMRs from cord blood (permutation *p*-value < 0.001), but farther than expected from DMRs in cortex (permutation *p*-value = 0.0001).

Despite ASD DMRs being closer together than expected by chance across many tissues, particularly in females, we hypothesized that newborn blood DMRs are largely tissue specific and would not differentiate ASD from TD samples from other tissues. To test this tissue specificity, we assayed the smoothed CpG methylation values in discovery newborn blood samples over the DMRs identified in cord blood, placenta, and cortex and confirmed by PCA that the samples did not separate by ASD diagnosis in either sex using DMRs from any tissue (Additional File 2, Figure S12).

### ASD DMRs identified from females are enriched for neurodevelopment-related biological processes in all tissues

**Fig. 5:**
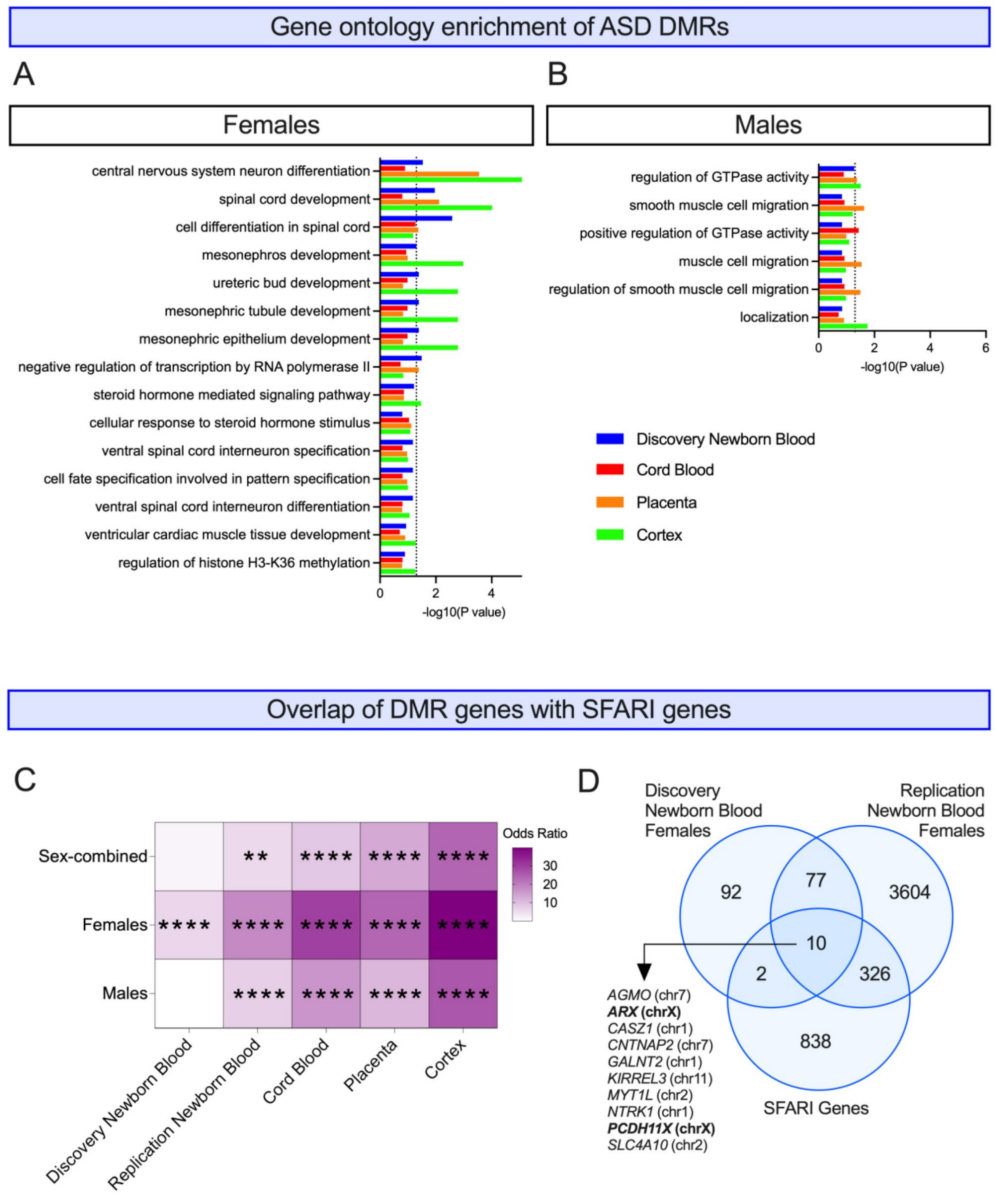
Enrichment of ASD DMRs with biological processes and ASD-risk genes. Gene ontology enrichment in **(A)** females and **(B)** males. Sex-specific DMRs from each tissue were examined for gene ontology enrichments using GOfuncR. Biological process enrichments with *p* < 0.2 from all tissues were overlapped and graphed by their -log(p-value). The dotted black lines represent *p* = 0.05. **(C)** Heatmap of sex-specific DMRs from each tissue overlapped with SFARI genes, with darker purple indicating a higher odds ratio (range: 0–40). * *p* < 0.05, ** *p* < 0.01, *** *p* < 0.001, **** *p* < 0.0001. **(D)** Venn diagram of DMR genes identified in females from discovery newborn blood and replication newborn blood overlapped with SFARI genes

We next performed gene ontology (GO) analysis on ASD DMRs from newborn blood as well as from cord blood, placenta, and cortex to evaluate whether consistent processes were epigenetically dysregulated across tissues. We selected all biological processes with *p* < 0.2 from each DMR comparison for overlap and found 15 terms that appeared in all tissues’ female DMR analyses, with the most significant term (on average across tissues) being “central nervous system neuron differentiation” (Fig. 5A). Other terms identified in females relevant to neurodevelopment or epigenetics included “ventral spinal cord interneuron specification” and “regulation of histone H3-K36 methylation”. In males, only six terms appeared in all tissues and were related to GTPase activity and muscle cell migration (Fig. 5B). 

### ASD DMR genes significantly overlap with SFARI genes, particularly in females

Because DMRs from all tissues were enriched for neurodevelopmental processes, we hypothesized that they were also enriched for known ASD-associated genes. To test this, we overlapped DMRs mapped to genes with all genes in the SFARI database [49]. In the discovery cohort newborn blood, only females were significantly enriched (*p* < 0.05) for SFARI genes, while in the replication cohort and all other tissues, females, males, and sex-combined DMRs were significantly enriched for SFARI genes (Fig. 5C; Additional File 1, Table S28).

To identify replicated newborn blood loci that have been genetically linked with ASD, we overlapped discovery and replication newborn blood DMR genes with SFARI genes. We found that of the 87 newborn blood DMR genes that replicated in females across two cohorts, 10 (11.5%) were listed as SFARI genes, two of which are X-linked (Fig. 5D): *AGMO* (chr7), *ARX* (chrX), *CASZ1* (chr1), *CNTNAP2* (chr7), *GALNT2* (chr1), *KIRREL3* (chr11), *MYT1L* (chr2), *NTRK1* (chr1), *PCDH11X* (chrX), and *SLC4A10* (chr2). No replicated DMRs genes identified from males were listed as SFARI genes.

## Discussion

In this study, we performed the first sex-stratified epigenomic signature of ASD from newborn blood spots, finding significant replication with an independent cohort, support for the female protective effect, and evidence of biological relevance of the gene loci and gene pathways identified. Our findings will help inform future efforts to develop newborn screening tools for ASD, improve our understanding of the etiology and pathology of ASD, and highlight the importance of sex-stratification in ASD studies.

Our ASD DMRs from discovery newborn blood revealed a signature of hypomethylation in ASD compared to TD samples as well as enrichment for DMR location in promoters, CpG islands, and CpG shores in both sexes, findings that have been previously reported in cord blood [18]. Despite the similarities in epigenetic profiles across the sexes, only one DMR gene was identified in all three comparisons (sex-combined, females only, and males only): *SPATA19* (Spermatogenesis Associated 19), which has been previously implicated in dysregulated neurology and behavior [51, 52]. *SPATA19* is predicted to be involved in sperm mitochondrion organization, and is essential for sperm motility and male fertility [53]. Based on gene ontology studies in Fragile X and Down syndrome human neural progenitor cells, *SPATA19* is involved in brain development, neurogenesis, and glial cell differentiation [54]. In a behavioral study of *Spata19* knockout mice, males showed altered behavior from wild-type males in several tests, including the social interaction and novel object recognition tasks [51]. Additionally, rare nonsense mutations in *SPATA19* were identified in affected siblings in an exome sequencing study of multiplex ASD families, indicating a possible convergence of genetic and epigenetic dysregulation in this gene [55]. While both sexes may share dysregulation in *SPATA19*, and this gene will be interesting to investigate in future studies, our findings indicate that most epigenetically dysregulated loci are sex-specific, demonstrating the value in sex-stratifying samples for ASD studies, despite reduced sample sizes.

Our analysis of sex differences in ASD provides the first epigenetic support for the female protective effect, which hypothesizes that the protective nature of certain factors in females (such as DNA methylation marks) require more changes to be attenuated to result in ASD, leading to fewer cases in females and stronger biological signatures. Our findings were most consistent with this model, and not reflective of the extreme male brain theory or gender incoherence theory, given that we found a stronger epigenetic signature of ASD in females over regions whose methylation levels differ by sex. We also found a striking overlap between female vs. male DMRs in ASD individuals and TD individuals, adding to the evidence that the mechanisms governing sex differentiation in neurotypical brains may also contribute to sex differences in ASD. It is notable that these biological differences are not only measurable in the brain but may also be reflected by methylation changes in newborn blood. The female protective effect is supported by neuroimaging studies, in which comparisons of ASD vs. TD individuals have shown larger differences in females than in males with regard to cortical thickness and development [56], organization of nerve fibers [57, 58], gray matter asymmetry [59], amygdala functional connectivity with the cortex [60], local connectivity within brain networks [61]. Similarly, genetic studies that have found that females with ASD carry more autism-associated genetic mutations than do males with ASD [62–66]. To our knowledge, this is the first DNA methylation study to examine proposed models for sex differences in ASD and report epigenetic support for the female protective effect. Additional studies are needed to confirm these findings.

These female-driven sex differences in ASD that we observed from autosomal DMRs may be augmented by the enrichment of female-specific ASD vs. TD DMRs on the X chromosome, which we observed in both cohorts of newborn blood. Because these X-linked genes were predominantly subject to X chromosome inactivation, we hypothesize a potential mechanism of X chromosome erosion in ASD females rather than a complete escape from repression on the inactive X chromosome, consistent with prior results [67]. Interestingly, enrichment for ASD DMRs on the X chromosome in both males and females has previously been reported in umbilical cord blood from a cohort with inherited enriched-likelihood for ASD [18]. Rather than reflecting a tissue difference, this finding may be due to differences in genetic risk for ASD (which is reflected in epigenetics) in the different cohort types. The discovery and replication newborn blood datasets from this study are from cohorts within the general population, while the cord blood was obtained from the MARBLES enriched-likelihood cohort, which enrolled pregnant women who already had a child with ASD [68]. Therefore, the MARBLES mothers may be protected carriers of genetic and/or epigenetic pre-disposition for ASDon the X chromosome, which then would likely have been reflected in enrichment for X-linked DMRs in both sexes. Further studies are needed to understand the inheritance patterns of X-linked DNA methylation alterations in multiplex ASD families.

Having established the sex specificity of the epigenetic signature of ASD, we hypothesized that sex-specific DNA methylation differences may be detectable across multiple perinatal tissues if the dysregulation occurred prior to tissue differentiation, lending insight to etiology and pathogenesis. In females, one or more DMRs from all four tissues mapped to *BCOR*, *GALNT9*, and *OPCML*, all of which have linked to neuropathology or dysregulated neurodevelopment [69–76]. It is striking that all three genes that replicated across tissues in females have roles in neurodevelopment, given that three out of four tissues are collected at the time of birth and are not directly linked to disease pathogenesis. *BCOR* encodes the BCL6 corepressor and plays a critical role in early embryonic development. A *BCOR* homolog, *BCORL1*, has been implicated in ASD in several studies [69–71]. *GALNT9* is part of the GALNT gene family that encodes enzymes involved in O-glycosylation; it is expressed specifically in the brain, most highly in the cerebellum, and its deletion has been previously implicated in ASD [74]. *OPCML* encodes neural cell adhesion molecules and SNPs in this gene have been linked to schizophrenia [75, 76]. *ZNF733P*, identified from all tissues in males, is a pseudogene located nearby many zinc-finger protein-encoding genes close to the centromere of chromosome 7 and to our knowledge, has not previously been associated with neurodevelopmental disorders.

We also found evidence for the biological relevance of the epigenetic signature of ASD in newborn blood and other perinatal tissues. Females in particular showed enrichment of DMRs from newborn blood, cord blood, placenta, and cortex for neurodevelopment-related biological processes, such as “central nervous system neuron differentiation”. In addition, ASD DMRs across all tissues and sexes were enriched for SFARI genes, indicating convergence of genetic and epigenetic markers of ASD and aligning with the findings from a 2021 study, in which autism-associated DNA CpG methylation sites from maternal blood, cord blood, and placenta samples were enriched for SFARI genes [21]. Overall, these results show the biological relevance of perinatal tissues as surrogates for the brain in understanding epigenetic dysregulation in ASD.

Despite its strength as the first WGBS study of ASD in newborn blood, this study was limited by its sample sizes. This is due the inherent challenge in obtaining perinatal samples from individuals later diagnosed with ASD, as well as ASD post-mortem samples. Consistent with most ASD studies, our cohorts have a strong male bias, which may influence the sex-specific findings. We down-sampled the males to address this limitation and found that results from subgroups of males were highly correlated with one another, increasing our findings in the results. We also addressed the inherent challenge of low sample sizes by using a replication cohort in newborn blood to reduce false positives. We anticipated that very few DMRs would replicate across cohorts, due to documented challenges in replicating DNA methylation biomarkers for ASD, including different study designs and technical factors, as well as different demographic factors, genetic backgrounds, and environmental exposures of the participants [77]. Despite these challenges, we found statistically significant replication of ASD DMRs in a separate cohort, as well as overlap with SFARI genes and DMRs identified in other ASD tissues. Because few overlaps were expected, we have greater confidence in the genes that were identified in multiple cohorts and/or tissues.

In addition to furthering the understanding of sex-specific epigenetic dysregulation in ASD, our results provide insights for biomarker development. Reproducible DNA methylation differences detectable in newborn blood could be potentially added to molecular screening panels to identify infants who should be behaviorally screened for ASD. Newborn biomarkers may be particularly useful for cases that are harder to identify behaviorally, and may help to overcome sex biases and other disparities in ASD diagnosis [78]. A positive screening result would inform parents and medical practitioners that diagnostic services will be paramount for the child and, importantly, a negative screening result would not exclude the possibility of future diagnosis. The possibility of using newborn blood for screening is particularly plausible given the accessibility and widespread collection of NDBS. While additional research is needed in this area, our study provides initial results supporting a possible future of ASD screening in newborns using DNA methylation from newborn blood.

## Conclusions

This study is the first to present sex-stratified WGBS DNA methylation signatures of ASD in newborn blood. We found a signature of DNA hypomethylation in ASD compared to TD samples in both sexes, and enrichment for DMRs in promoters, CpG islands and CpG shores. Because there was little overlap between DMR genes identified from females and males, we further explored sex differences in newborn blood and are the first to our knowledge to report epigenetic support for the female protective effect. We further found that the newborn blood ASD DMRs overlapped with those from other perinatal tissues (umbilical cord blood and placenta) as well as post-mortem cortex, showing that despite the tissue-specificity of DNA methylation there may be some loci that are consistently dysregulated across tissues, perhaps due to disruption early in life. ASD DMRs from all tissues were enriched for neuro-related biological processes (females) and SFARI ASD genes (females and males), showing the biological relevance of the dysregulated loci and the convergence of genetic and epigenetic markers for ASD. This study presents a step forward in identifying potential biomarkers of ASD in newborns as well as providing insights to sex-specific epigenetics dysregulation in ASD.

## Electronic supplementary material

Below is the link to the electronic supplementary material.

Supplementary Material 1

Supplementary Material 2

## Data Availability

Code for this study is available on GitHub (https://github.com/juliamouat/NewbornBlood_DNAmethylation_ASD). The WGBS data from cord blood, placenta, and cortex are available in the Gene Expression Omnibus with accession numbers GSE140730 (cord blood), GSE178206 (placenta), GSE81541 (cortex) and GSE119981 (cortex). The WGBS data from the discovery and replication newborn blood are not publicly available: Any uploading of genomic data (including genome‐wide DNA methylation data) and/or sharing of California Biobank Program biospecimens or individual data derived from these biospecimens has been determined to violate the statutory scheme of the California Health and Safety Code Sect. 124980(j), 124991(b), (g), (h), and 103850 (a) and (d), which protect the confidential nature of biospecimens and individual data derived from biospecimens. Should we be contacted regarding individual-level data contributing to the findings reported in this study, inquiries will be directed to the California Department of Public Health Institutional Review Board to establish an approved protocol to utilize the data, which cannot otherwise be shared peer-to-peer.
