# Supplementary material for: Sex-specific DNA methylation signatures of autism spectrum disorder from whole genome bisulfite sequencing of newborn blood
Source: Biol Sex Differ. 2025 Apr 30;16:30. doi: 10.1186/s13293-025-00712-9 (PMC12042393; doi:10.1186/s13293-025-00712-9)
Supplement: Supplementary file 1 [file 13293_2025_712_MOESM1_ESM.pdf]

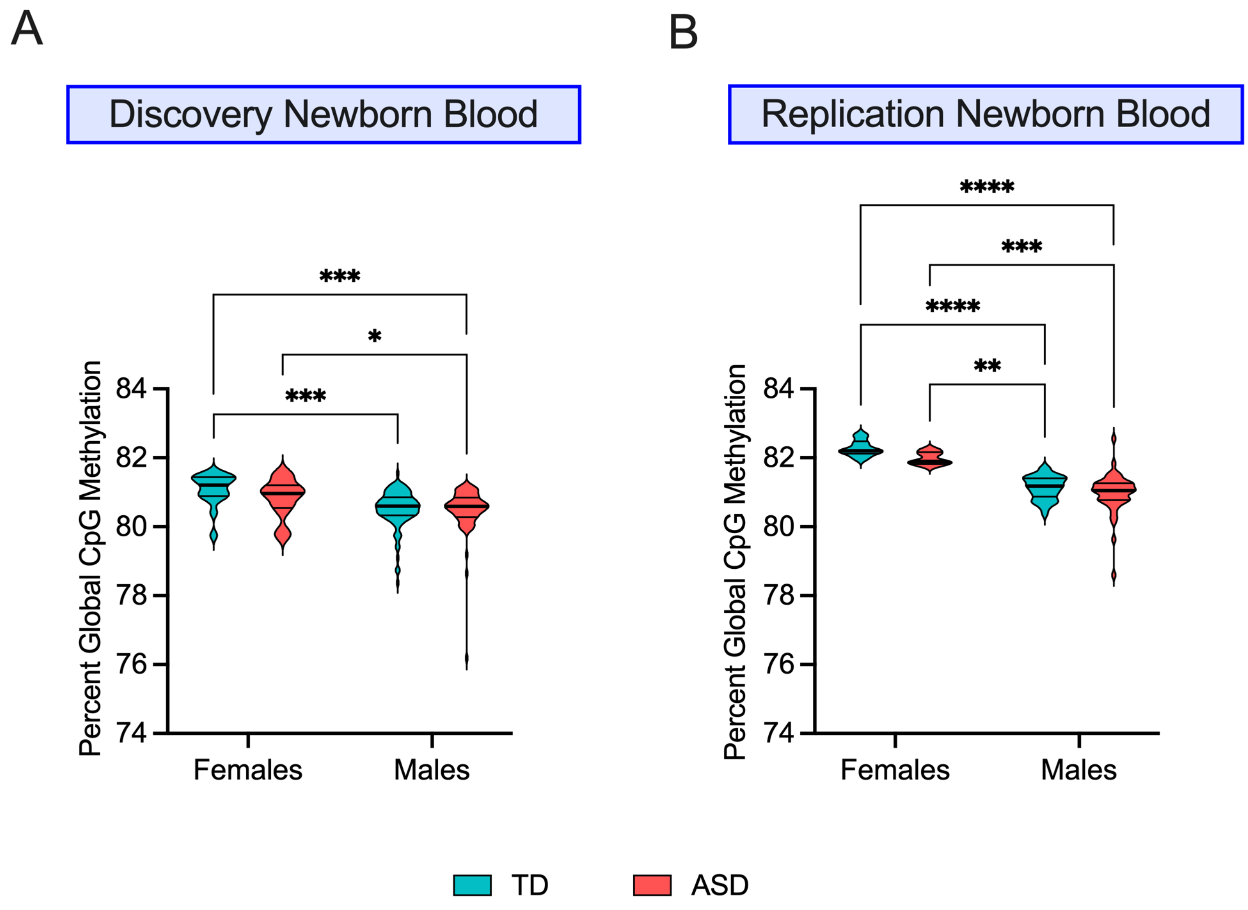

Supplemental Figure 1. Percent global CpG methylation in **A)** discovery and **B)** replication newborn blood, separated by sex and ASD diagnosis. Significance was tested using two-way ANOVA with Fisher's Least Significant Difference test for individual comparisons (* p <0.05, ** p <0.01, *** p <0.001, **** p <0.0001).

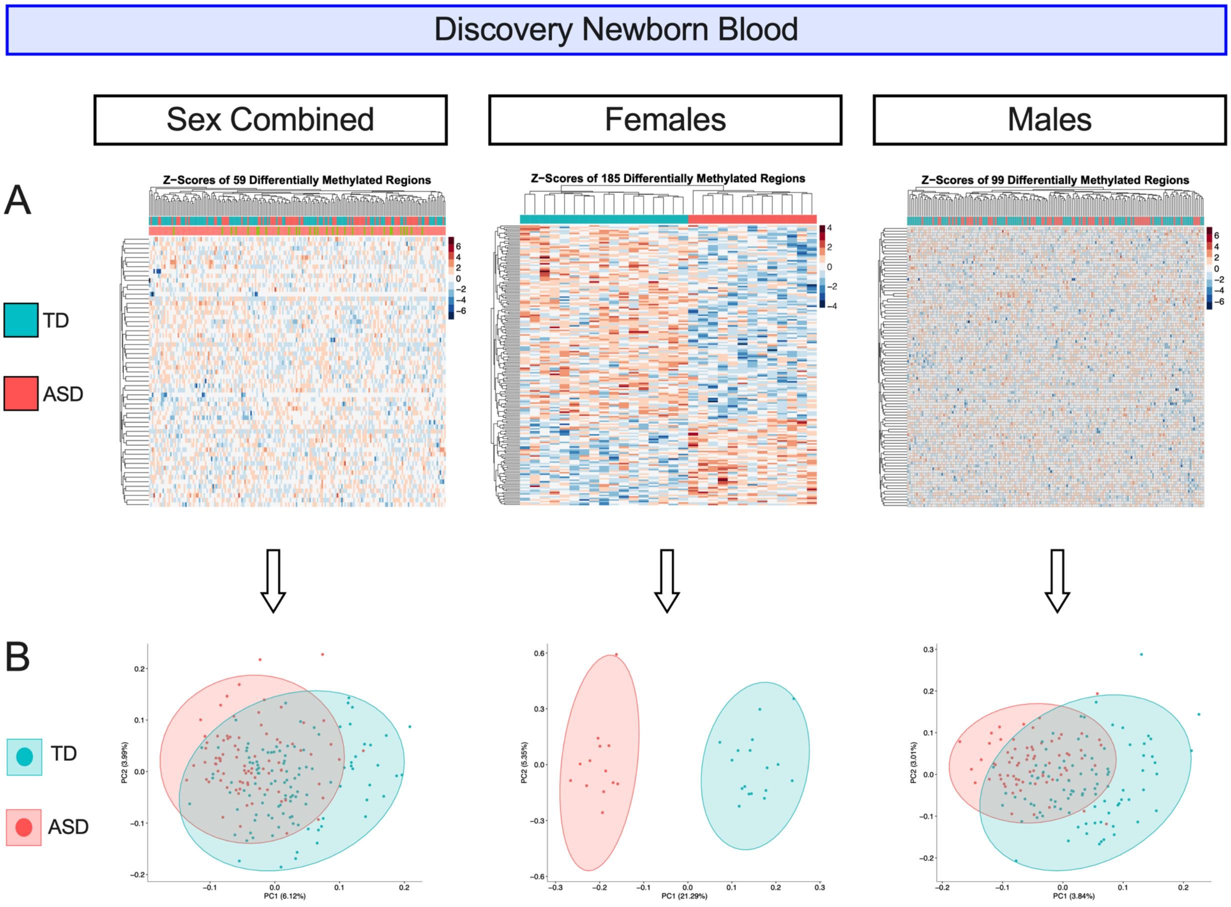

Supplemental Figure 2. Differentially methylated regions from sex-combined, females only, and males only comparisons of ASD vs TD samples in discovery newborn blood. **A)** Heatmaps of nominally significant ($p < 0.05$) DMRs, which are hierarchically clustered by their Z-scores, the number of standard deviations from the mean smoothed methylation value for each DMR. **B)** PCA plots of smoothed methylation values of the DMRs, colored by ASD diagnosis. The semi-transparent circles on the PCA plots represent 95% confidence intervals.

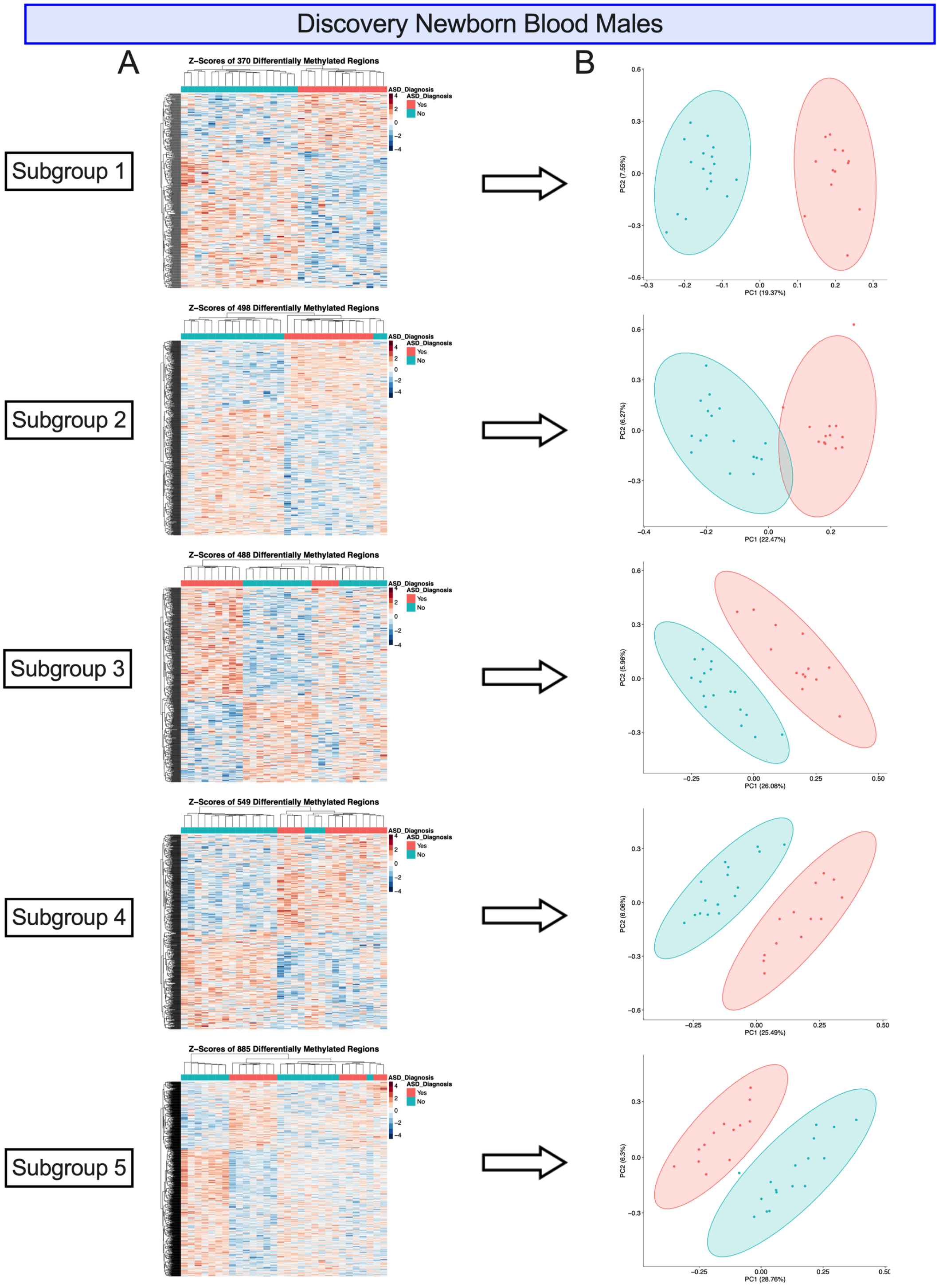

Supplemental Figure 3. Differentially methylated regions from discovery newborn blood ASD vs TD males. Each subgroup contains 30 randomly selected, non-repeated samples (17 TD, 13 ASD) to match the number of discovery newborn blood females. **A)** Heatmaps of nominally significant (p < 0.05) DMRs, which are hierarchically clustered by their Z-scores, the number of standard deviations from the mean smoothed methylation value for each DMR. **B)** PCA plots of smoothed methylation values of the DMRs, colored by ASD diagnosis. The semi-transparent circles on the PCA plots represent 95% confidence intervals.

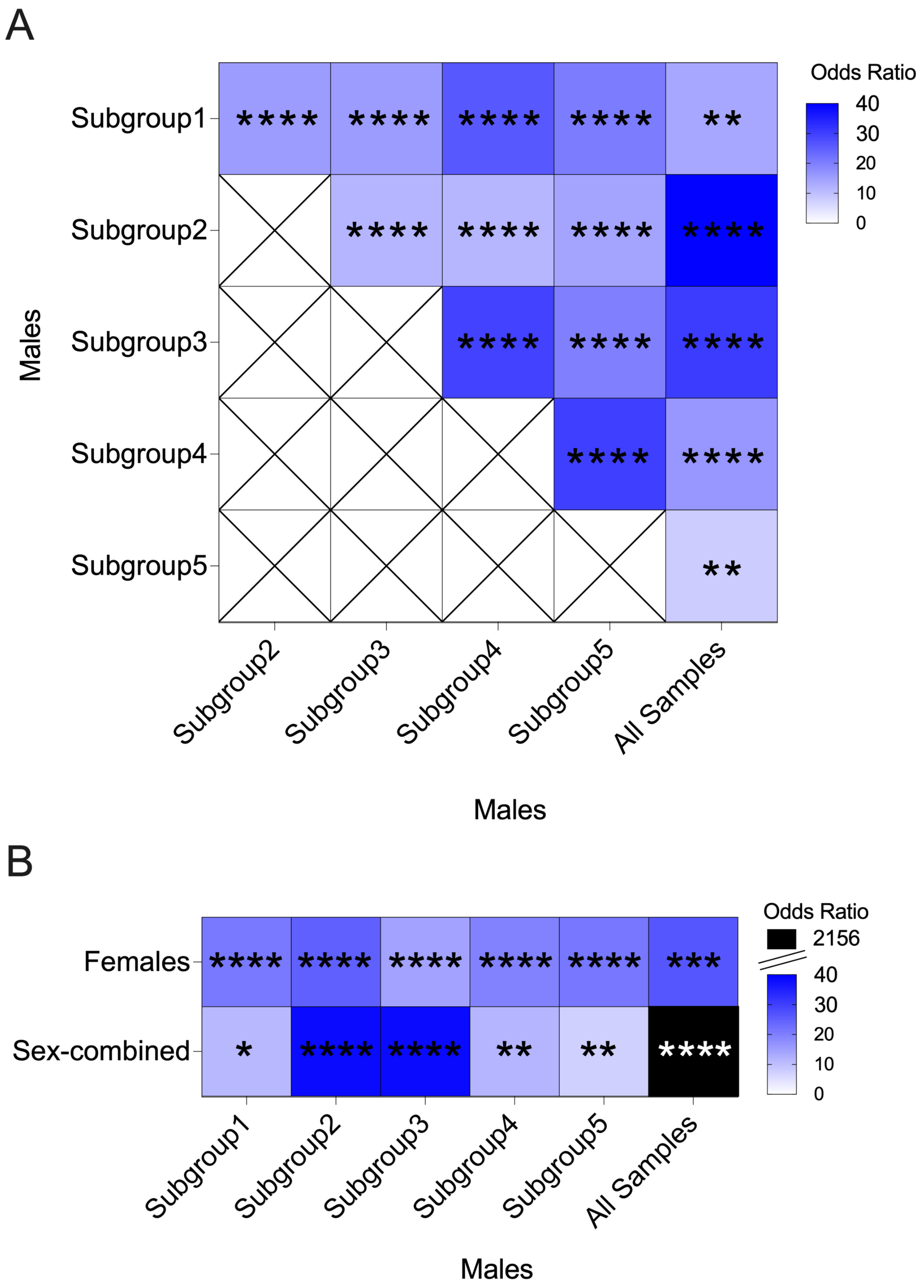

Supplemental Figure 4. Odds ratios of overlaps of discovery newborn blood ASD DMR gene names from the five subgroups each containing 30 males with **A)** one another and the ASD DMRs from all males and **B)** with ASD DMRs from the females only and sex-combined comparisons. Heatmap shows odds ratios of overlaps where darker blue indicates a higher odds ratio. * p <0.05, ** p <0.01, *** p <0.001, **** p <0.0001.

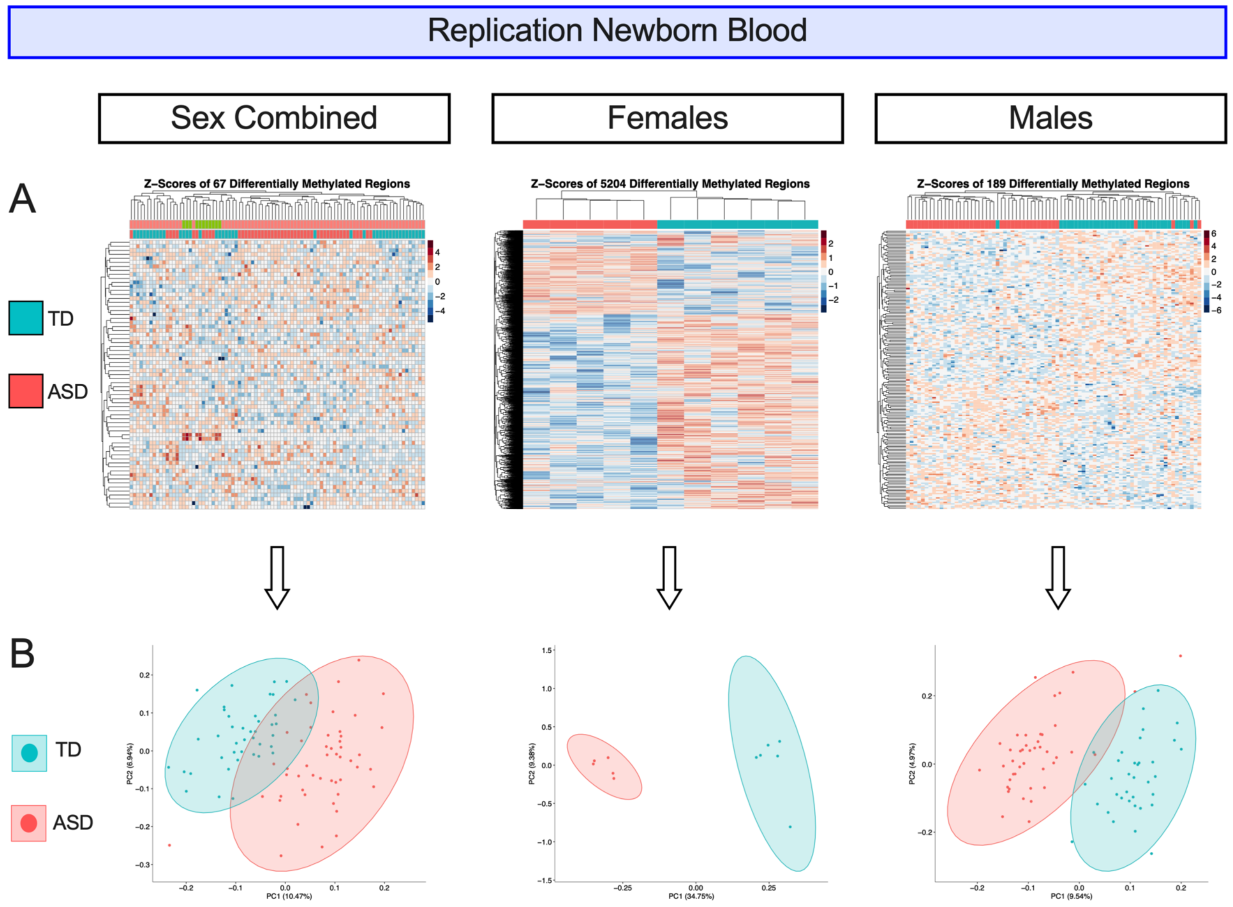

Supplemental Figure 5. Differentially methylated regions from sex-combined, females only, and males only comparisons of ASD vs TD samples in replication newborn blood. **A)** Heatmaps of nominally significant ($p < 0.05$) DMRs, which are hierarchically clustered by their Z-scores, the number of standard deviations from the mean smoothed methylation value for each DMR. **B)** PCA plots of smoothed methylation values of the DMRs, colored by ASD diagnosis. The semi-transparent circles on the PCA plots represent 95% confidence intervals.

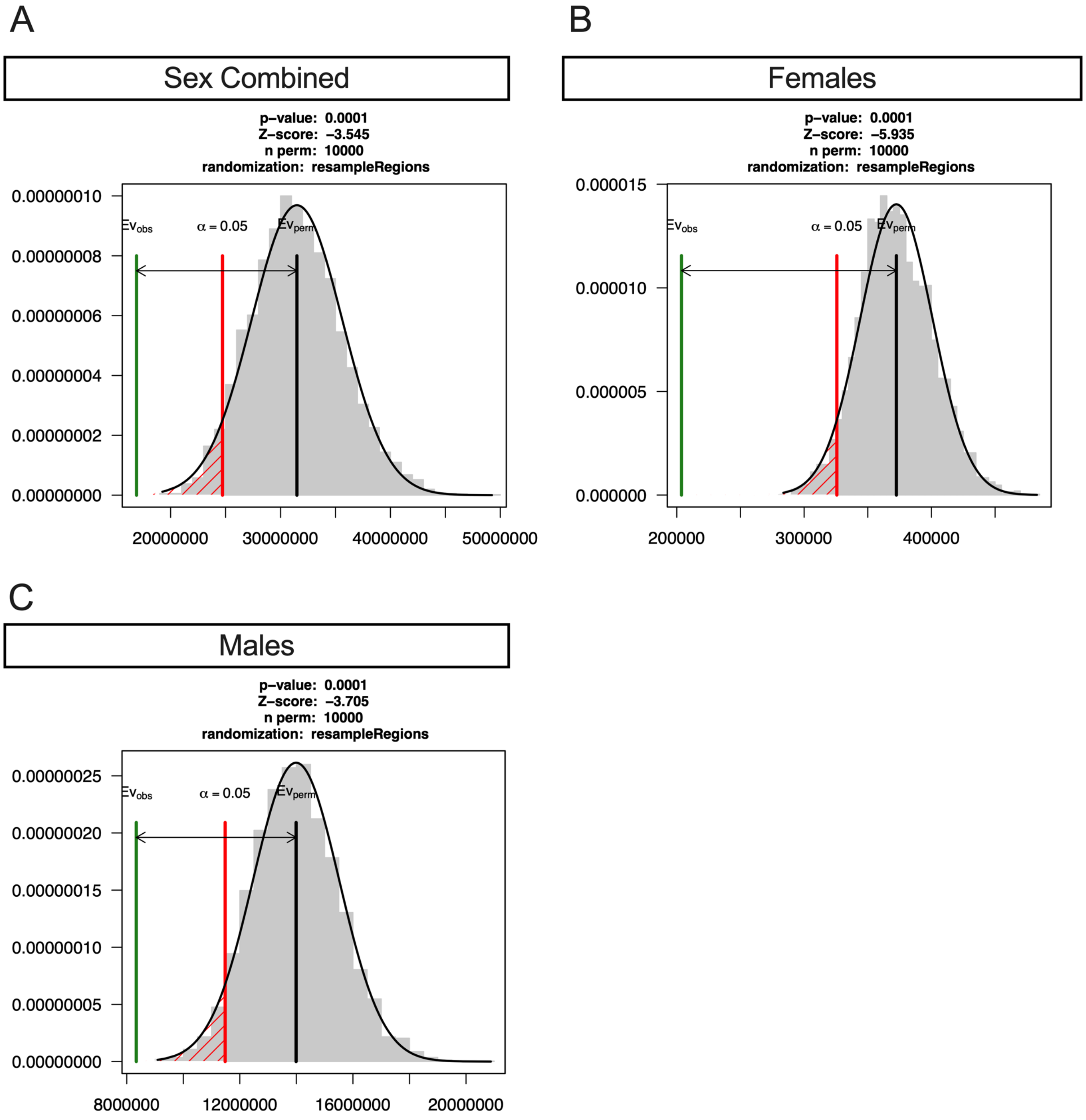

Supplemental Figure 6. Pairwise comparisons of the proximity of any given ASD DMR in discovery newborn blood to the closest ASD DMR in replication newborn blood in **A)** sex-combined, **B)** females only and **C)** males only comparisons using 10,000 permutations of randomly resampling regions. The black line is the expected distance, the red line is alpha = 0.05, and the green line is the observed distance.

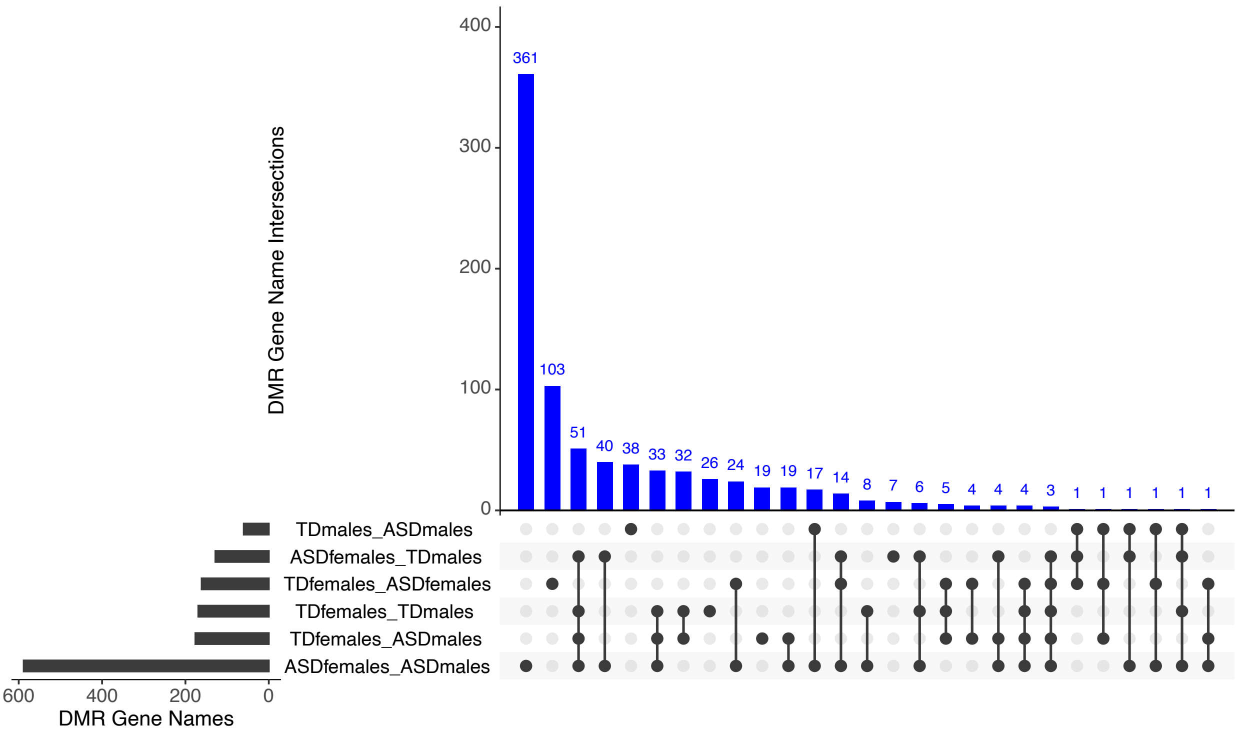

Supplemental Figure 7. UpSet Plot of autosomal DMRs in discovery newborn blood intersected by gene name.

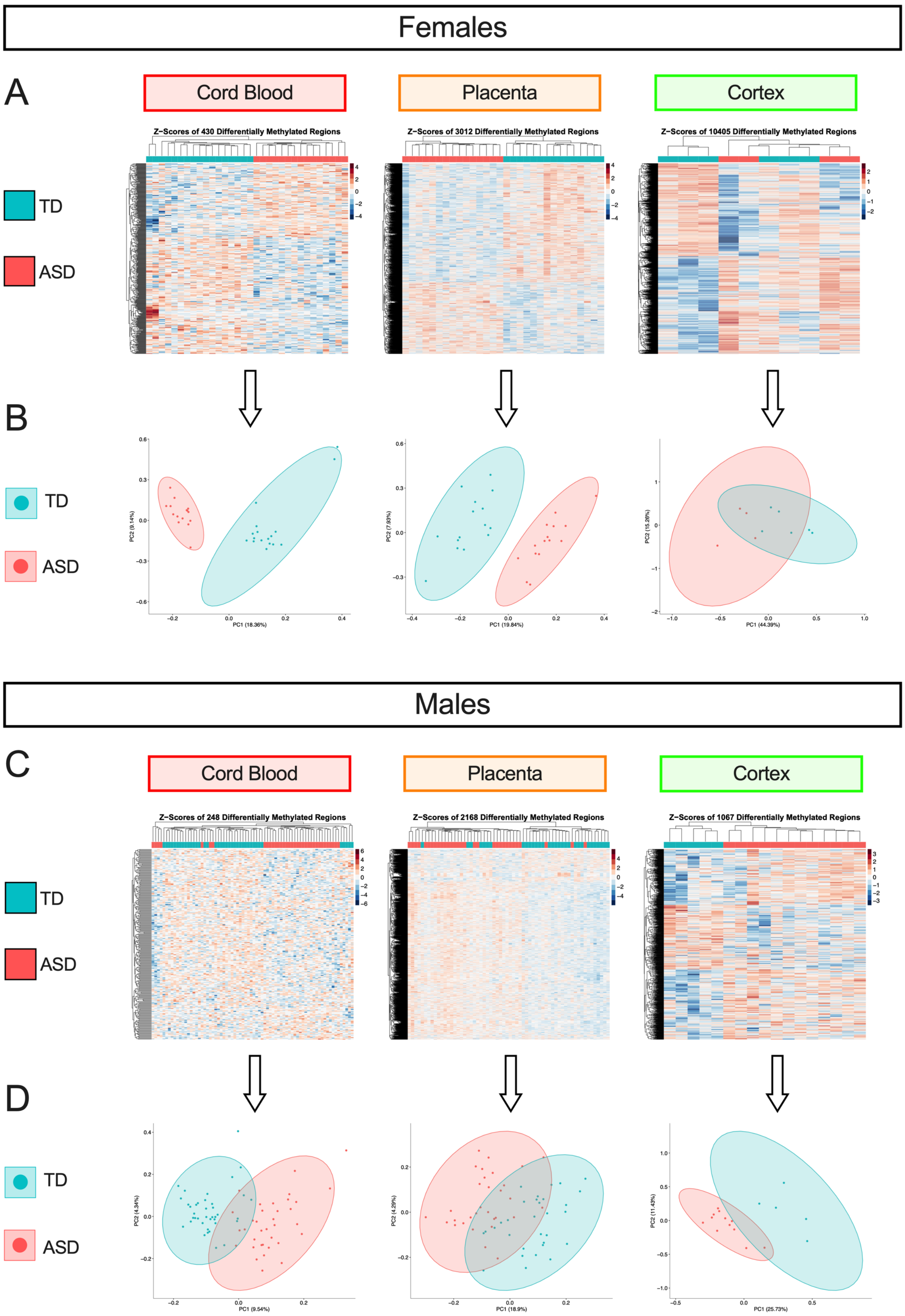
Females
A
Cord Blood
Placenta
Cortex
Z−Scores of 430 Differentially Methylated Regions
Z−Scores of 3012 Differentially Methylated Regions
Z−Scores of 10405 Differentially Methylated Regions
TD
ASD
B
TD
ASD
PC1 (18.36%)
PC2 (9.14%)
PC1 (19.84%)
PC2 (7.93%)
PC1 (44.39%)
PC2 (15.26%)
Males
C
Cord Blood
Placenta
Cortex
Z−Scores of 248 Differentially Methylated Regions
Z−Scores of 2168 Differentially Methylated Regions
Z−Scores of 1067 Differentially Methylated Regions
TD
ASD
D
TD
ASD
PC1 (9.54%)
PC2 (4.34%)
PC1 (18.9%)
PC2 (4.29%)
PC1 (25.73%)
PC2 (11.43%)

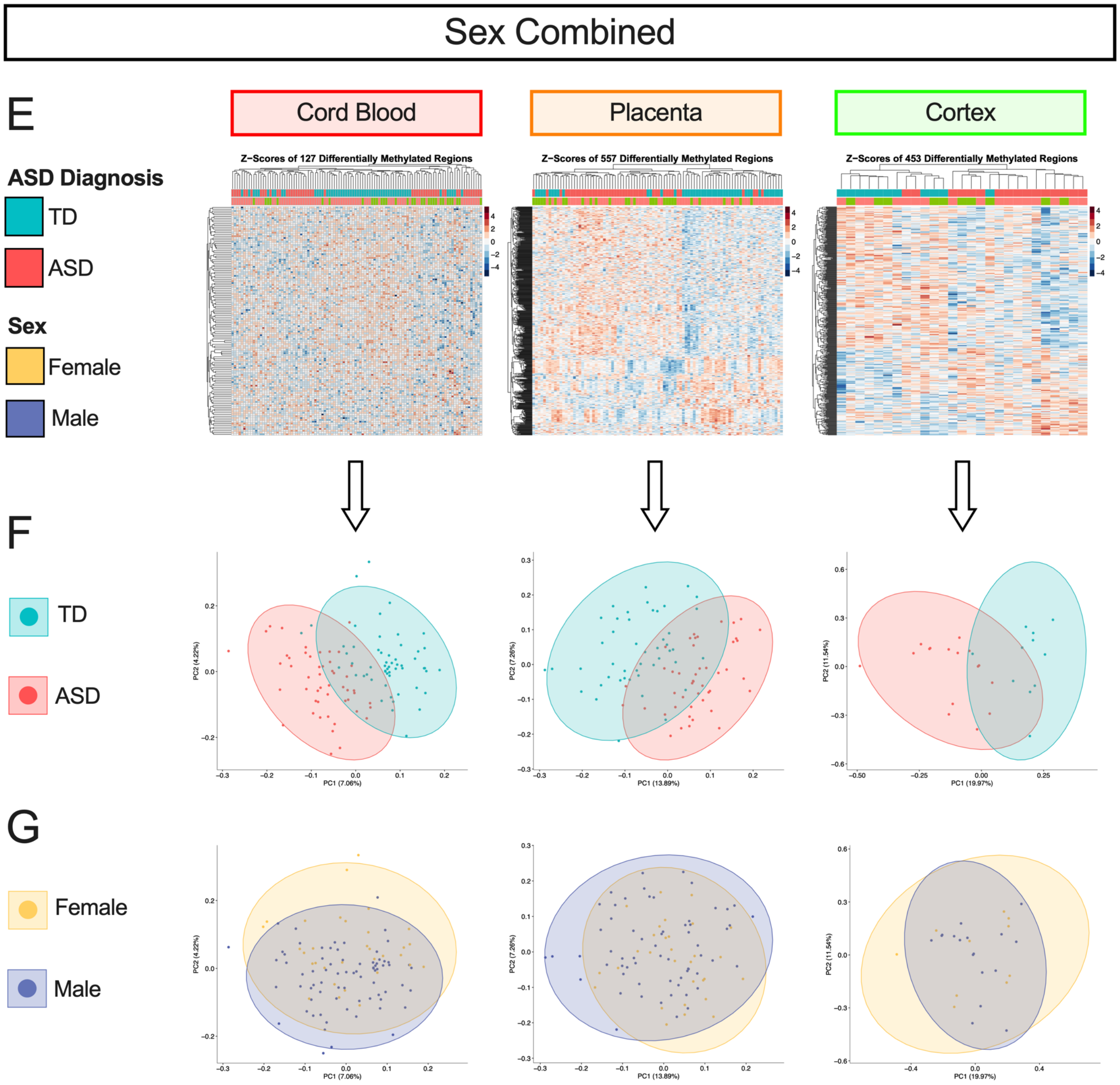

Supplemental Figure 8. Differentially methylated regions from ASD vs TD samples in cord blood, placenta, and cortex in females only, males only, and sex-combined (corrected for sex) comparisons. **A)** From females only, heatmaps of nominally significant (*p* <0.05) DMRs that are hierarchically clustered by their Z-scores, which are the number of standard deviations from the mean smoothed methylation value for each DMR. **B)** From females only, PCA plots of smoothed methylation values of the DMRs, colored by ASD diagnosis. The semi-transparent circles represent 95% confidence intervals .**C)** same as (A) in male samples. **D)** same as (B) in male samples. **E)** same as (A) in sex-combined samples. **F)** same as (B) in sex-combined samples. **G)** same as (B) in sex-combined samples except the PCA plots are colored by sex.

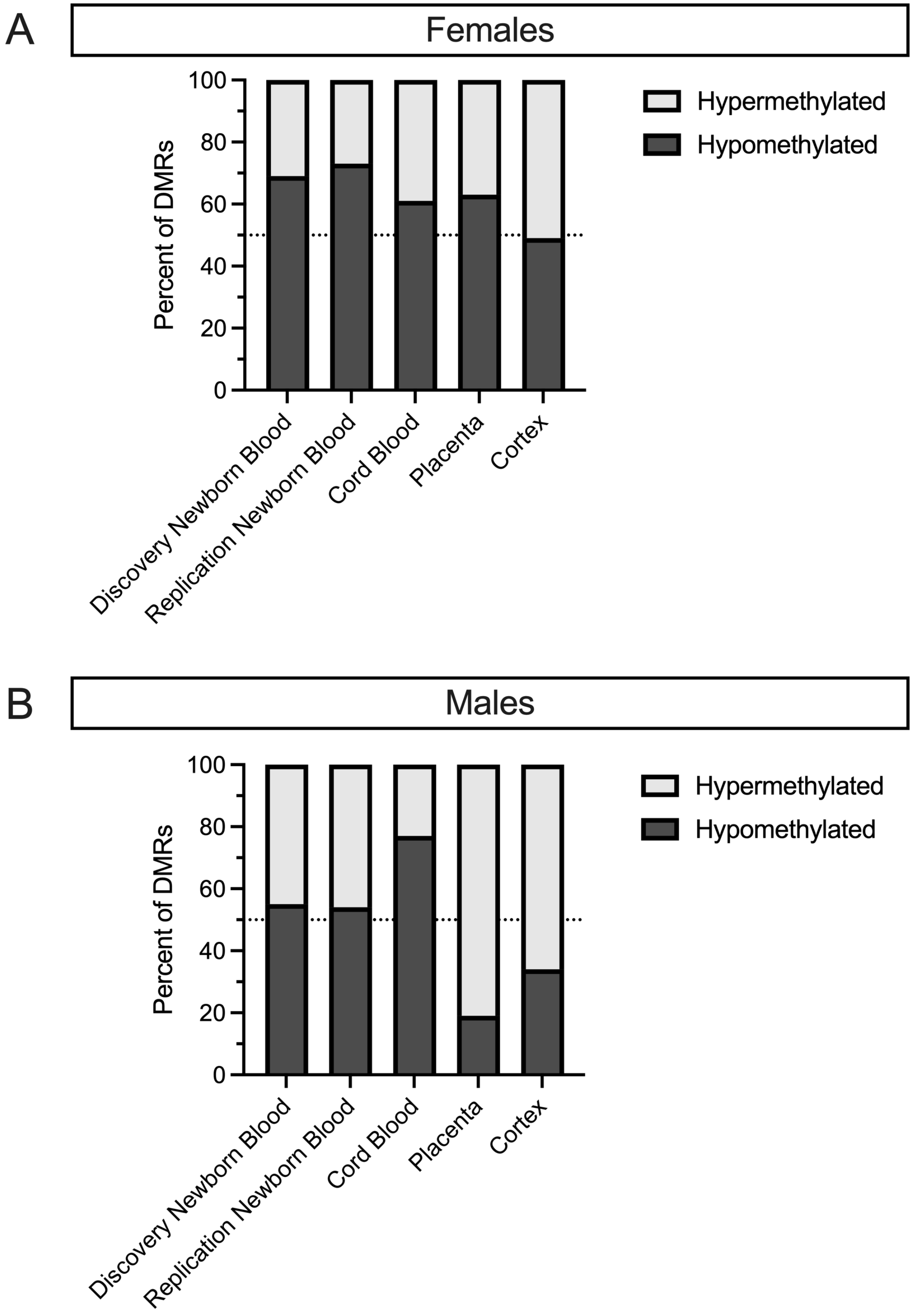

Supplemental Figure 9. The percent of ASD DMRs that are hypermethylated/hypomethylated in ASD vs TD samples in **A)** females and **B)** males across all tissues.

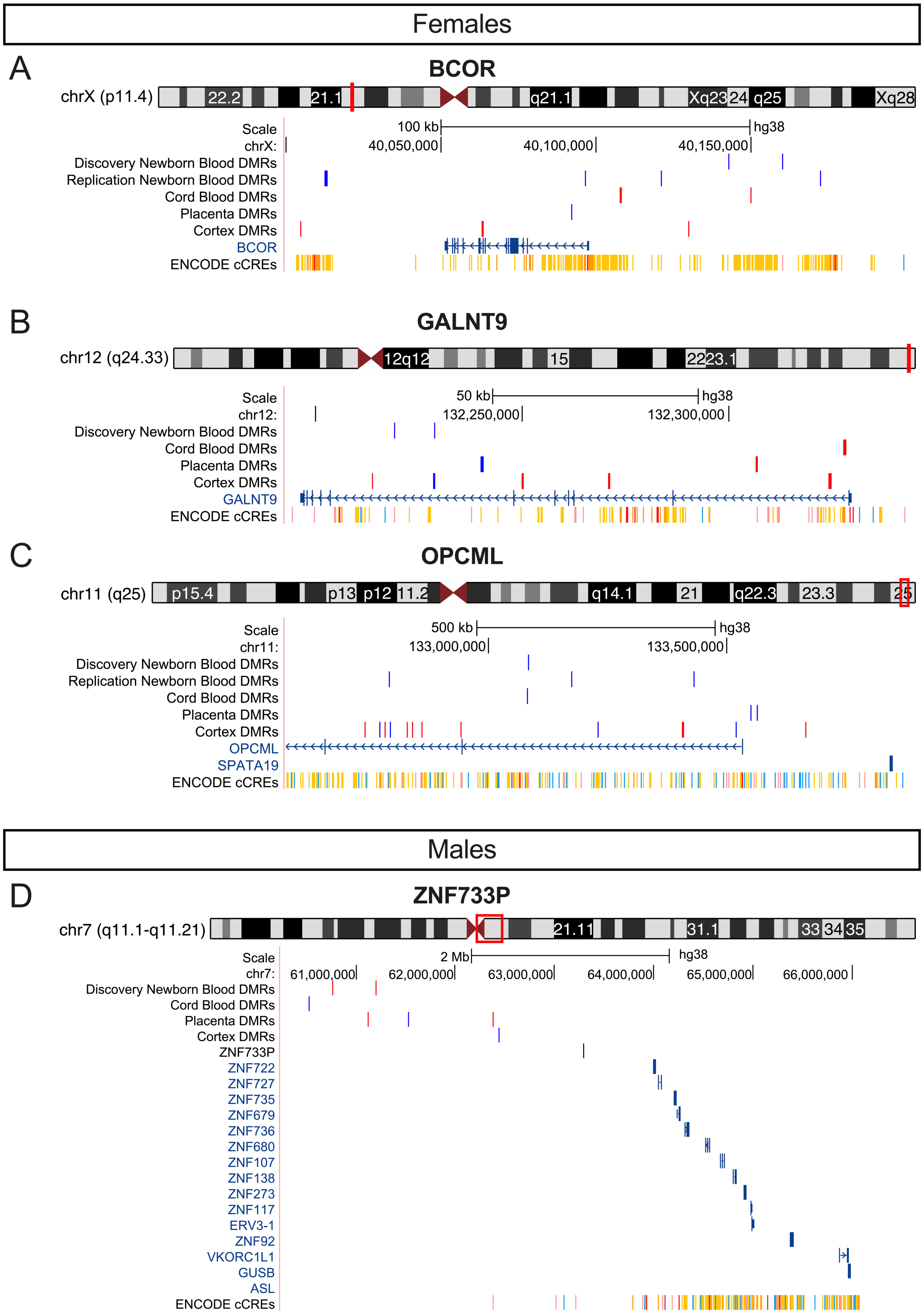

Supplemental Figure 10. **UCSC Genome Browser showing location of DMRs from all tissues** that mapped to **A)** *BCOR*, **B)** *GALNT9*, **C)** *OPCML*, and **D)** *ZNF733P*. Red lines indicate that the DMR was hypermethylated in ASD compared to TD samples with blue lines indicate hypomethylated DMRs.

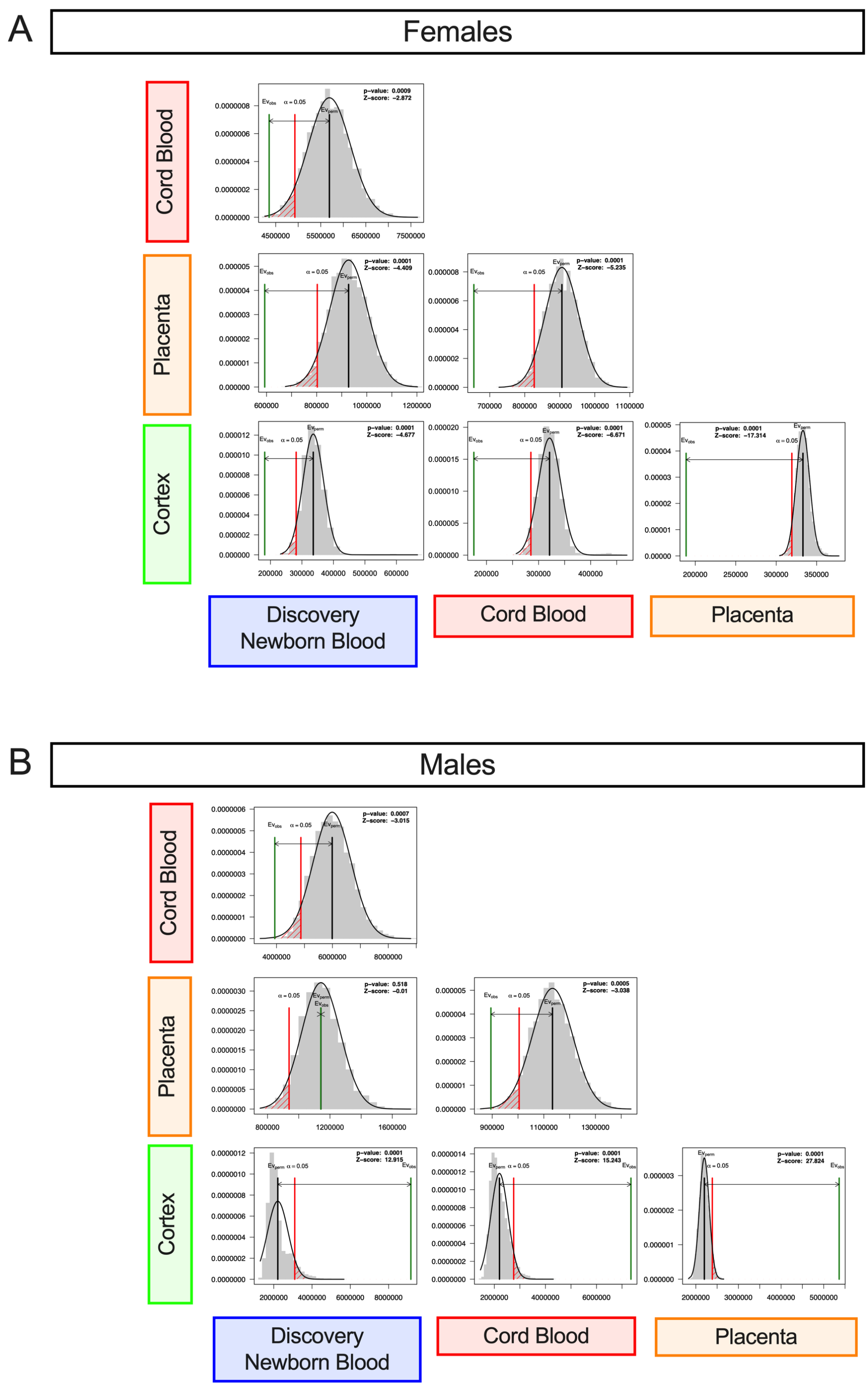

Supplemental Figure 11. Pairwise comparisons of the proximity of any given DMR to the closest DMR in another tissue in **A)** females and **B)** males using 10,000 permutations and random resampling of regions. The black line is the expected distance, the red line is alpha = 0.05, and the green line is the observed distance.

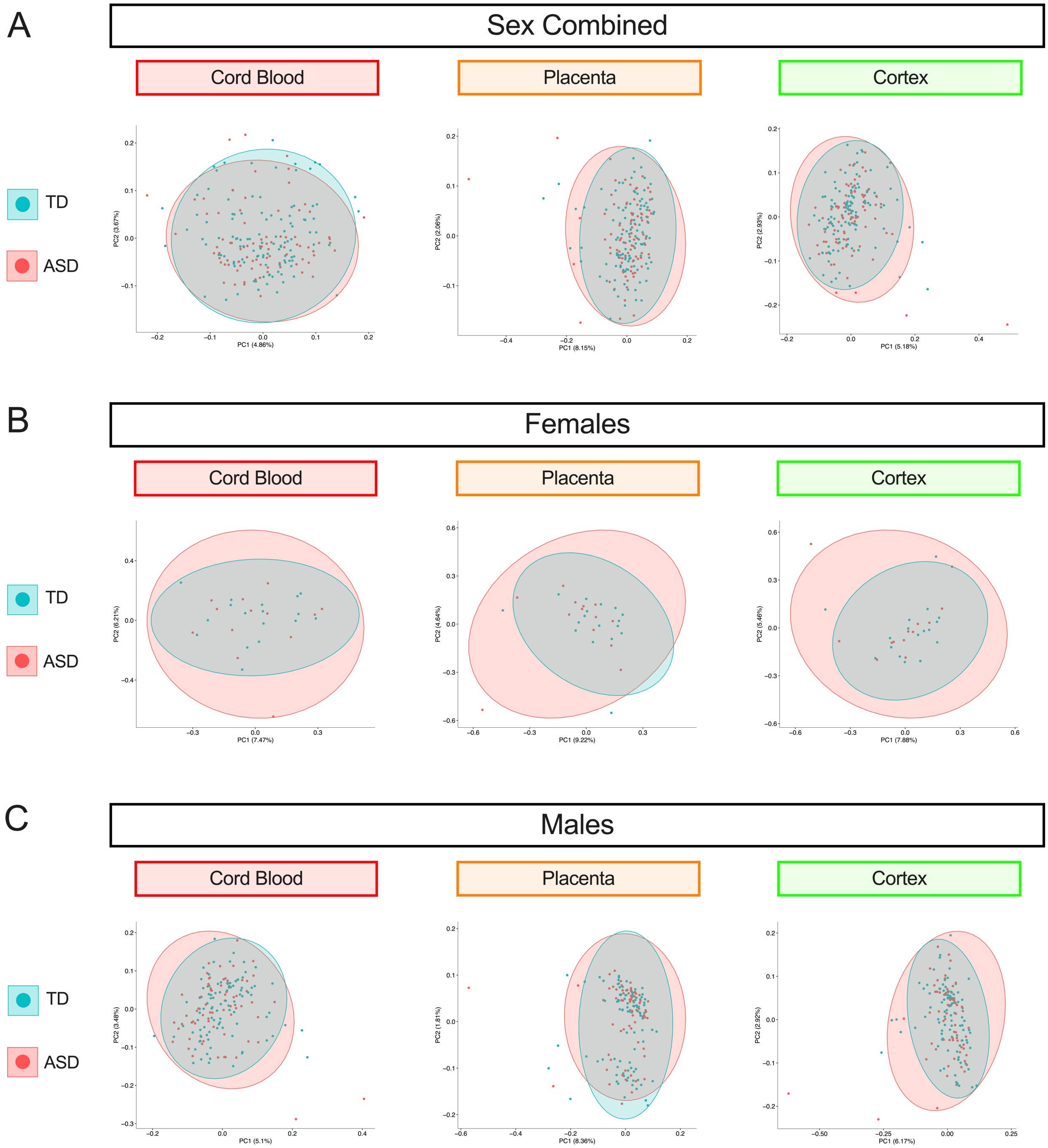

Supplemental Figure 12. PCA plots of smoothed methylation values in discovery newborn blood over DMRs identified from cord blood, placenta, and cortex in **A)** sex-combined, **B)** females only, and **C)** males only comparisons. The semi-transparent circles on the PCA plots represent 95% confidence intervals.
